# Screening pertactin-specific antibodies and evaluating competitive epitope recognition by native mass spectrometry

**DOI:** 10.1039/d5sc09702a

**Published:** 2026-03-17

**Authors:** Mohamed I. Gadallah, Kate A. McConnell, Kelli M. Hager, Virginia K. James, Annalee W. Nguyen, Jennifer A. Maynard, Jennifer S. Brodbelt

**Affiliations:** a Department of Chemistry, The University of Texas at Austin Austin TX 78712 USA jbrodbelt@cm.utexas.edu; b Department of Pharmaceutical Analytical Chemistry, Faculty of Pharmacy, Assuit University Assuit 71526 Egypt; c Department of Molecular Biosciences, The University of Texas at Austin Austin TX 78712 USA; d Department of Chemical Engineering, The University of Texas at Austin Austin TX 78712 USA

## Abstract

Structural characterization of antigen–antibody interactions is critical for understanding protective vaccine responses and development of therapeutic monoclonal antibodies (mAb). Traditional biophysical and biochemical techniques often require the immobilization of one binding partner or provide ensemble-averaged measurements, constraints which may limit the ability to probe multiple facets of antigen–antibody interactions. Native mass spectrometry (nMS) offers a versatile alternative, providing a comprehensive view of antigen–antibody complexes. Here, we utilized native MS to screen the interactions between a small panel of monoclonal antibodies (mAbs) and the *Bordetella pertussis* vaccine antigen mature pertactin (Prn), offering in-depth characterization of binding affinity, stoichiometry, and competition. We implemented variable temperature electrospray ionization to evaluate thermally induced unfolding and stability of different mAb·Prn complexes, while biolayer interferometry (BLI) and competition experiments were employed to provide complementary information about binding kinetics and mapping of distinct epitopes on Prn. Finally, we used nMS to evaluate the interactions of individual mAbs with Prn variants as a predictor for therapeutic action. Our results demonstrate the utility of nMS in combination with other techniques as a powerful approach for understanding the interactions of protective mAb binding to Prn, providing insight into mechanisms of vaccine-induced protection.

## Abstract 2

Structural characterization of antigen–antibody interactions is critical for understanding protective vaccine responses and development of therapeutic monoclonal antibodies (mAb).

## Graphical abstract

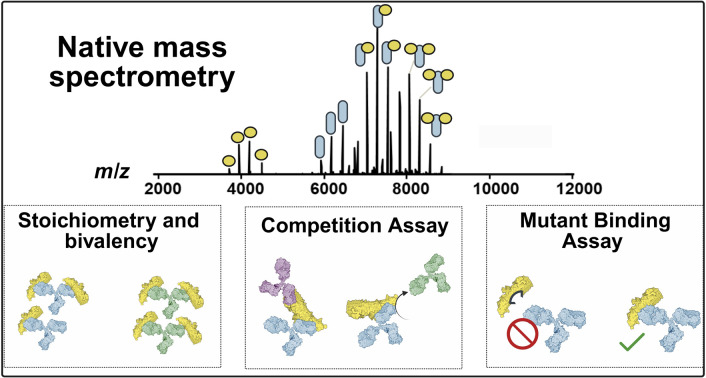

## Introduction

Antibodies are a crucial component of the protective immune response elicited by many vaccines to prevent severe viral and bacterial infections, including SARS-CoV-2 and *Bordetella pertussis*.^1–3^ Due to their conserved beta-sheet structure with six hypervariable loops, antibodies can form complementary geometric and electrostatic surfaces that allow specific binding to secreted or cell-surface antigens. Protection against whooping cough, a respiratory disease caused by the Gram-negative bacterium *Bordetella pertussis*, is mediated by antibodies against a variety of antigens.^4–7^ In the US and much of the world, acellular pertussis vaccines comprise a cocktail of two or more purified antigens, including pertussis toxin (Ptx), filamentous hemagglutinin (Fha), fimbriae 2 and 3, and pertactin (Prn).^4^ Unlike immune responses to Ptx^8–13^ and Fha,^14^ Prn is the only current acellular vaccine antigen that elicits bactericidal antibodies.^15^ While many circulating *B. pertussis* strains have lost Prn expression, structurally homologous autotransporter proteins, including BrkA, Vag8, and BapC, are under consideration for inclusion in next-generation vaccines.^15^ Accordingly, increased understanding of antibody/Prn interactions as a model system can provide insights into antigenicity, bacterial responses to immune pressures, and protection that are likely relevant for all *B. pertussis* autotransporter proteins, which themselves represent potential vaccine targets in the event of pertactin escape.

**Fig. 1:**
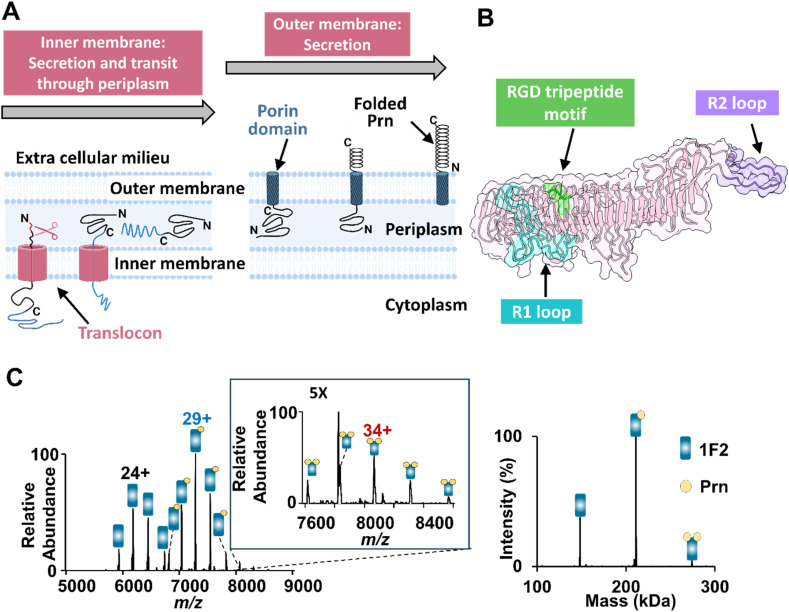
Prn is a target for bactericidal antibodies that prevent pertussis infection. (A) Schematic illustrating the process of Prn secretion and folding. Pertactin is initially expressed in the cytoplasm as a precursor protein with an N-terminal signal peptide (red), which directs it to the Sec pathway for translocation into the periplasm. In the periplasm, signal peptidases cleave the signal peptide, allowing further maturation. Subsequently, the porin domain (30 kDa) is embedded in the outer membrane, followed by secretion of the passenger domain. Finally, the passenger domain is cleaved yet remains non-covalently attached to the bacterial outer membrane. (B) Crystal structure of mature Prn antigen (1DAB) with 16 parallel β-helices forming the backbone of the protein. Highlighted within this structure are several key regions: the R1 loop (cyan color), which can be involved in antigen recognition; the RGD motif (depicted in green), a possible site for interacting with host cellular receptors;^16^ and the C-terminal R2 loop in purple, which plays a role in protein–protein interactions. (C) ESI mass spectrum obtained for a solution containing 2 µM 1F2 antibody and 2 µM Prn in 250 mM ammonium acetate with 0.06% of C10E5. The deconvoluted mass spectrum confirming the formation of both 1F2·Prn and 1F2·[Prn]_2_ complexes is shown on the right.

Pertactin is a classical autotransporter with unclear function, although it has been speculated to contribute to bacterial adhesion by virtue of a solvent-exposed RGD motif. It is expressed as a precursor protein with two domains (Fig. S1): a C-terminal porin domain (30 kDa) which forms a beta-barrel membrane protein that transports the N-terminal passenger domain (63 kDa) across the outer membrane. Once on the cell surface, Prn is cleaved to release the mature passenger domain, which remains non-covalently associated with the bacterial outer membrane (Fig. 1A).^16^ Mature Prn is a β-helical protein comprising 16 parallel right-handed β-helices (Fig. 1B) with several protruding loops.^16,17^ This includes proline-rich regions with tandem repeat sequences: the R1 loop contains one to three repeats of (GGXXP), while the R2 loop contains two to eight (PQP) repeats across *Bordetella* species.^18^ Vaccination with whole-cell vaccines was associated with changes in the repeat sequences while vaccination with pertactin-containing acellular vaccines appears to have driven loss of Prn expression,^19^ leading to the speculation that these regions may be immunodominant and may contribute to bacterial evasion.

Antibody-mediated protection depends on non-covalent interactions with an antigen, with features such as binding affinity, epitope recognition and valency playing important roles.^20,21^ Accordingly, characterizing these interactions is key to understanding antibody biological activities and can guide the design of immunogens intended to preferentially elicit protective antibodies.^22^ Biolayer interferometry (BLI)^23^ and surface plasmon resonance (SPR)^24^ provide rapid and accurate measurements of antigen–antibody binding kinetics and affinities, but offer limited information regarding binding stoichiometry and interaction interfaces. High-resolution methods such as X-ray crystallography^25^ and cryogenic electron microscopy (cryo-EM)^26^ can resolve atomic-level details of protein–protein interactions, but are typically limited to analysis of the monovalent fragment antigen-binding (Fab) region to avoid structural heterogeneity from the dynamic hinge region.^27–29^ As a result, these methods do not capture the effects of steric hindrance or cooperativity associated with full-length antibodies.^27,30^ Mass spectrometry-based protein footprinting methods such as fast photochemical oxidation of proteins (FPOP)^31,32^ or hydrogen–deuterium exchange mass spectrometry (HDX-MS) have been widely used for epitope and/or paratope mapping,^32–38^ providing complementary insights into antibody–antigen interfaces.^33,39^ Size-exclusion chromatography multi-angle light scattering (SEC-MALS) can measure aggregation and molecular weight distributions of antibody–antigen complexes in solution.^40–42^ However, the outcomes reflect bulk molecular weight based on the hydrodynamic volume rather than accurate masses, making this approach less effective at resolving closely related species, deconvoluting complex mixtures, or detecting glycoform heterogeneity.^40,41^ Consequently, understanding the full scope of antibody–antigen interactions necessitates combining various biophysical and analytical methods.

Native mass spectrometry (nMS) is a well-established analytical technique for characterizing intact proteins and their non-covalent complexes under native-like solution conditions, and recent advances continue to expand its applications and scope of insight.^43–45^ For nMS, proteins are prepared in solutions containing mM concentrations of volatile salts, such as ammonium acetate, to increase the ionic strength of the solutions relative to conventional denaturing MS. Electrospray ionization (ESI) is employed to transfer the proteins and/or protein complexes into the gas phase. Native MS can provide detailed structural information, including subunit stoichiometry, in addition to insight into protein structural dynamics and binding kinetics.^43,46^ For antibodies and other biologicals, nMS serves as a powerful tool to reveal information on primary structure, oligomerization, and degradation.^47–49^ With its exceptional mass accuracy and high resolution, nMS pushes the boundaries of mass analysis, enabling the detailed study of interactions between therapeutic mAbs and different antigen proteoforms.^50^ Moreover, results from nMS can be combined with data from other techniques to gain deeper insights into antibody structural dynamics and stability.^49,51^ For instance, coupling nMS with ion mobility (IM) allows the study of stability and separation of antibody structural isoforms as well as antibody–antigen complexes, enabling the measurement of collision cross sections and structural topology.^52–54^ Additionally, variable temperature electrospray ionization (vT-ESI) can be employed to evaluate the thermal stability and structural unfolding of antibodies and their complexes.^55^ Recent comparative analyses have further underscored the complementarity of nMS with SEC-MALS, native charge-detection MS, and mass photometry for the comprehensive characterization of antibodies and heavily glycosylated immune complexes.^51^ The integration of nMS with other biophysical techniques has the potential to provide a holistic view of the antigen–antibody binding landscape. This multifaceted approach can facilitate deconvolution of antibody responses to vaccine antigens and support antibody discovery campaigns to identify those binding discrete epitopes.

Here, we utilized nMS with other methods to characterize the interactions of a panel of six monoclonal antibodies with the extracellular region of the Prn antigen. Combining these techniques provides comprehensive information about antibody binding kinetics, the stability and specificity of antibody–antigen interactions, and identifies antibodies binding different epitopes.

## Results and discussion

### Screening of Prn-specific monoclonal antibodies using native MS

Evaluating the epitope binding of mAbs or even the antibodies elicited by therapeutic vaccines is crucial, as antibody efficacy hinges on its ability to bind to a specific target and, often, a specific epitope on that target. Therefore, the ability to screen and characterize antigen–antibody interactions is essential for the development, optimization and evaluation of antibodies. This assessment typically requires several chemical and biophysical techniques to decipher all facets of binding affinities, stoichiometries and stabilities of the antigen–antibody complexes. Here, we utilize nMS to characterize the interactions of a set of mAbs with mature Prn. The mature Prn antigen is a peripheral outer-membrane protein that requires the presence of membrane mimetics such as detergent micelles to achieve adequate solubilization and stabilization for electrospray ionization and transport into the gas phase.^56^

The proteins must be released from the detergent micelles prior to mass analysis; this is generally accomplished using mild collisional activation such as in-source trapping to simultaneously disrupt the micelles and avoid protein unfolding or dissociation of non-covalent complexes.^57^ Hence, we screened the solubilization of Prn using four nMS-compatible detergents including (*n*-dodecyl β-d-maltoside (DMM), *n*-octyl-beta-d-glucopyranoside (OG), pentaethylene glycol monodecyl ether (C10E5) and tetraethylene glycol monooctyl ether (C8E4)) and determined the optimum desolvation voltage required for disassembly of different detergent micelles with minimum protein unfolding (Fig. S2). Our findings suggest that glycol detergents, specifically C10E5 and C8E4, allowed effective detergent removal at a relatively low in-source trapping voltage of −10 V (Fig. S2).

Based on ESI-MS, the charge state distribution for Prn in solution with glycol detergents centered around 16+ (*m*/*z* 3933) with minor detergent adducts. Increasing the in-source trapping voltage up to −50 V more efficiently released Prn from the micelles and shed bound detergents with no evidence of significant protein unfolding (based on no observable change in the charge state distributions). In contrast, ESI of Prn in solution with non-ionic detergents DDM and OG required higher in-source trapping voltages (−90 to −100 V) to achieve similar results. With OG, a broadening of the charge state distribution was observed, shifting the most abundant charge state to 17+ at *m*/*z* 3702. Of all the detergents tested, C10E5 demonstrated superior performance, requiring a lower desolvation voltage and a lower critical micelle concentration of only 0.06%, compared to 0.25% for C8E4 (Fig. S3).^58^ Hence, C10E5 was selected as a solubilizing detergent for different Prn variants (wild type and mutants) (Fig. S4) for the remainder of the study.

Native MS offers a high throughput method for screening interactions between mAbs and Prn without labeling or immobilization steps. To leverage this capability, the monoclonal antibodies used in this study were selected based on a prior study demonstrating that anti-pertactin mAbs define four groups corresponding to spatially distinct epitopes on Prn.^59^ One representative antibody with high apparent affinity was chosen from each group (2E9, 1F2, 2B1, and 1E7). For the initial MS screening experiments, solutions containing mature Prn or each mAb were buffer-exchanged in 250 mM ammonium acetate (with the addition of 0.06% C10E5 detergent for the Prn antigen only). Prn was incubated with each mAb at an equimolar ratio (2 µM each) for one hour at room temperature followed by MS analysis. Initially, we probed the interactions between Prn and either 1F2 as a specific mAb that binds Prn with high affinity or a non-binding control mAb (NC). For the non-binding isotype mAb, no complexes with Prn were observed (Fig. S5A), confirming the inability of NC to interact with Prn. In contrast, 1F2 produced two types of complexes with Prn (Fig. 1C), one corresponding to abundant 1 : 1 1F2·Prn complexes with an average charge state centered around the 29+ charge state (∼210 kDa) (Table S1) and the other consistent with very low abundance 1 : 2 complexes, *i.e.*, 1F2·[Prn]_2_ (32+ to 36+ charge states, 272 kDa). Screening of the solutions containing Prn and the other body-binding mAbs showed similar formation of both 1 : 1 and 1 : 2 mAb·Prn complexes (Fig. S5). The high signal intensities observed for both the 1 : 1 and 1 : 2 mAb·Prn complexes in these nMS experiments suggest that these mAbs have high affinities for the Prn antigen. Despite their nanomolar affinities, full occupancy was not observed for the 1F2, 2B1, and 1E7 mAbs. This can be explained by partial dissociation of mAb·Prn complexes during ionization and transmission in the mass spectrometer, as well as differences in relative ESI response factors. Free antibodies often ionize and transmit more efficiently than large complexes, which can skew the apparent abundances observed in nMS. To account for differences in ionization and transmission efficiencies, we attempted to implement a slow mixing mode (SLOMO) nanoESI-MS strategy, a time-resolved continuous solution mixing method designed to enable estimation of dissociation constants independent of relative ESI response factors.^20^ However, quantitative determination of dissociation constants using SLOMO was not feasible in this case, likely due to the slow diffusion rate of the large Prn antigen (Fig. S6).

Consistent with the nMS results, biolayer interferometry (BLI) measurements also demonstrated strong binding of all four antibodies (1E7, 2B1, 1F2, and 2E9) to Prn (Fig. S7). The BLI association and dissociation profiles are characteristic of high-affinity interactions and align closely with the nanomolar dissociation constants previously reported for these antibodies in the literature.^59^ These complementary observations support the conclusion that the complex formation observed by native MS reflects high-affinity recognition of Prn.^59^

### Differential bivalent engagement revealed by native MS

**Fig. 2:**
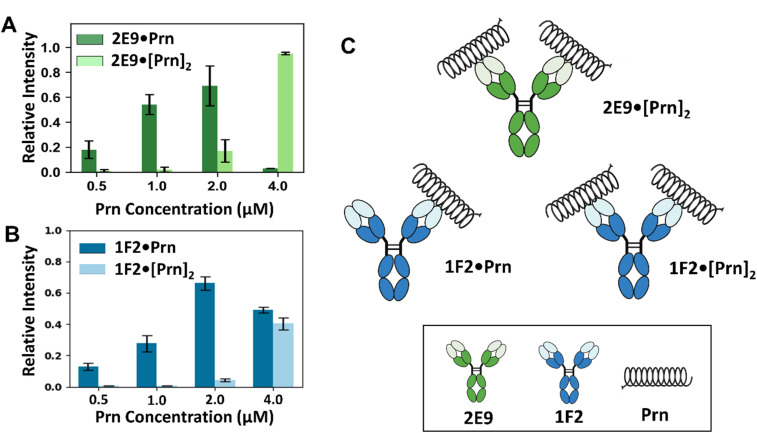
Evaluation of antibody–antigen binding stoichiometry by native MS. (A and B) Relative intensities of the 1 : 1 and 1 : 2 mAb·Prn complexes obtained from titrations of 2 µM (A) 2E9 or (B) 1F2 with increasing Prn concentrations (0.5–4 µM) in 250 mM ammonium acetate containing 0.06% C10E5. (C) Schematic representations of predominant binding configurations for 2F9 (top) and 1F2 (bottom). All antibodies can form 1 : 1 and 1 : 2 complexes; however, 2E9 shows a higher fraction of 1 : 2 species at elevated Prn concentrations, consistent with enhanced bivalent engagement.

Ab·Prn complex stoichiometry is important for elucidating function and mechanism of action, directly influencing the response kinetics and activity threshold of therapeutic mAbs and serving as a pivotal determinant in their functional efficacy.^60^ While several biophysical techniques can characterize antigen–antibody binding specificity and kinetics, few of them provide detailed stoichiometric information. To address this, we employed nMS, which allows direct measurement of the molecular weights of intact complexes in the gas phase, offering high resolution and accuracy, thereby revealing precise stoichiometries without the need for labeling or immobilization. For nMS, we performed titration experiments using solutions containing a fixed concentration of an mAb (2 µM) and a variable concentration of Prn (0.5 µM to 8 µM), while monitoring the resulting mass spectra. Examples of the MS1 spectra, the deconvoluted spectra, and graphs displaying the relative abundances of mAb·Prn and mAb·[Prn]_2_ complexes are shown in Fig. S8–S11 for 2E9, 1F2, 2B1, and 1E7, respectively. As the concentration of Prn increased, the relative abundances of the 1 : 1 mAb·Prn complexes increased. When the Prn-to-mAb ratio exceeded 1 : 1, the abundances of 1 : 2 mAb·[Prn]_2_ complexes increased significantly. The relative distribution of 1 : 1 and 1 : 2 complexes, however, differed across antibodies. All four mAbs showed concentration-dependent increases in the mAb·[Prn]_2_ complexes, yet the extent of this shift varied. For 1F2, 2B1, and 1E7 (Fig. 2B, S10B and S11B), the distribution of mAb·Prn and mAb·[Prn]_2_ complexes remained at comparable levels throughout the titration, suggesting similar propensities for single- and dual-antigen engagement. In contrast, 2E9 displayed a pronounced increase in the 1 : 2 mAb·[Prn]_2_ complex at higher Prn concentrations (Fig. 2A), indicating a greater tendency for bivalent binding through both Fab arms. These results demonstrate that although all four mAbs can accommodate two Prn molecules, the extent to which both Fab arms participate varies among antibodies, reflecting differences in epitope accessibility or Fab flexibility. The schematic in Fig. 2C and the deconvoluted MS data (Fig. S12) highlight these distinct binding configurations, with 1F2 representing a mixed population of 1 : 1 1F2·[Prn] and 1 : 2 1F2·[Prn]_2_ complexes and 2E9 favoring the bivalent 1 : 2 species. Importantly, increasing the Prn concentration to 8 µM did not result in higher-order complex formation (Fig. S8–S11), indicating that the maximum stoichiometry achieved at micromolar concentrations was 1 : 2 mAb·[Prn]_2_. Moreover, neither 2 : 2 [mAb]_2_·[Prn]_2_ nor 2 : 1 [mAb]_2_·Prn complexes were observed, confirming that each mAb binds to only one binding site on Prn.

### Epitope overlap and dual antibody binding revealed by nMS

Pertactin is the only current vaccine antigen that elicits bactericidal antibodies; these likely act through antibody-dependent phagocytosis or antibody-dependent complement cytotoxicity, immune activities which require Fc clustering for activation. This readily occurs when multiple antibodies simultaneously bind adjacent epitopes on the same bacterial surface antigen.^8,10^ Traditionally, screening for simultaneous binding of antibodies to antigens has been performed using BLI experiments or cryo-EM.^23,61^ These methods, however, require either the immobilization of one of the interaction partners^23,61^ or the utilization of only the Fab region to avoid the inherent flexibility and dynamic nature of the intact mAb molecule.^30^ In contrast, nMS experiments provide an alternative option to screen for the simultaneous binding of mAbs using the intact Abs. This approach allows the consideration of binding competition and steric hindrance that arise from the full-length molecule, thereby capturing a more accurate picture of the actual simultaneous binding process.^27^

**Fig. 3:**
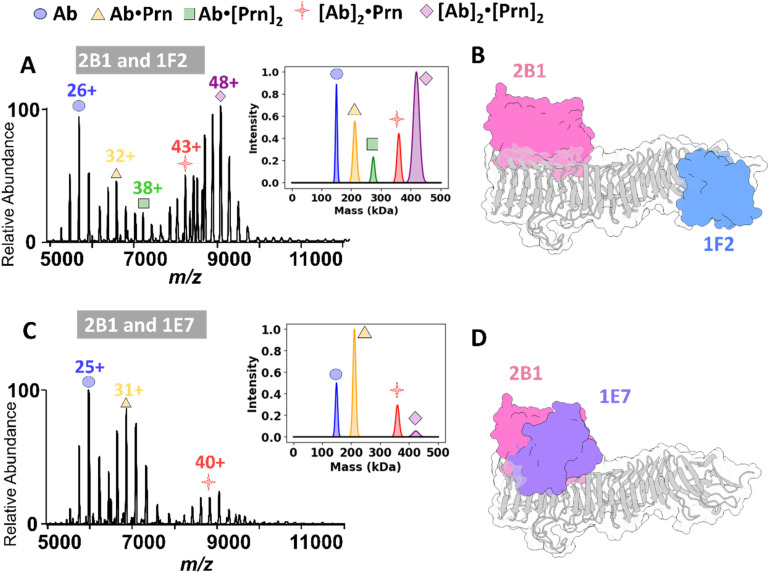
Binding competition experiments between different pairs of antibodies interacting with the Prn antigen. (A and C) MS1 spectra obtained from incubation of 2B1 (5 µM) with either 1F2 (5 µM) or 1E7 (5 µM) and Prn (5 µM), with deconvoluted spectra shown in the insets on the right. (B and D) Schematic representations of Prn + 2B1-Fab + 1F2-Fab and Prn + 2B1-Fab + 1E7-Fab, respectively, with Prn shaded gray (1DAB), and AlphaFold-predicted structures for 2B1-Fab shaded pink, 1F2-Fab shaded blue and 1E7-Fab shaded purple, all shown as molecular surfaces. The model in 3D displays the potential overlap of 2B1 and 1E7 that would impede simultaneous binding. The presence of high intensity ion peaks centered at *m*/*z* 9000 corresponds to complexes containing Prn and two different antibodies that bind to distant epitopes such as 2B1 and 1F2 and demonstrates that two antibodies can simultaneously bind to the Prn antigen. Conversely, simultaneous binding of antibodies that bind to overlapping or proximate epitopes (2B1 and 1E7) is suppressed, resulting in low intensity peaks for the [Ab]_2_·[Prn]_2_ complexes.

BLI competition data from a previous study showed that the antibodies 1E7, 2B1, 1F2, and 2E9 each recognized distinct Prn epitopes.^59^ However, this method did not fully resolve how closely these epitopes are positioned or whether antibody pairs experienced steric interference when binding simultaneously. To investigate this issue, we used nMS to examine the ability of selected full-length mAb pairs to simultaneously bind Prn. This approach allowed us to distinguish between antibodies targeting overlapping or spatially constrained epitopes and those binding distant, non-competing regions. For these experiments, 5 µM of each of two full-length antibodies was incubated with 5 µM of Prn, followed by acquisition of ESI mass spectra to profile the complexes. For antibody pairs targeting distinct non-overlapping epitopes on Prn (*e.g.*, 2B1/1F2, 1E7/2E9, 2B1/2E9 and 1E7/1F2), ion peaks consistent with Ab·Prn, Ab·[Prn]_2_, and very abundant [Ab]_2_·[Prn]_2_ complexes were observed (Fig. 3A and S13A–C). The [Ab]_2_·[Prn]_2_ ion peaks correspond to the formation of complexes in which two full-length Abs bind simultaneously to two Prn molecules, forming large complexes (∼420 kDa). The [Ab]_2_·Prn complexes are particularly interesting because they confirm two Abs bound to a single Prn antigen and presumably should only be possible when the epitopes are sufficiently spaced apart such that the antibodies can bind concurrently without competitive or steric interference, resulting in stable complexes.

For three of these mAb pairs (2B1/1F2, 1E7/2E9, and 1E7/1F2), low abundance [Ab]_2_·Prn complexes were detected, but not for 2B1/2E9. The [Ab]_2_·[Prn]_2_ complexes were extremely dominant for the 2B1/2E9 pair, perhaps obscuring the deconvolution and assignment of the [Ab]_2_·Prn complexes in Fig. S13B. To aid in visualization, Fig. 3B and S13D–F show the previously reported crystal structure of Prn (1DAB)^17^ with AlphaFold-predicted structures of different Fab fragments bound at illustrative positions. These models were generated by manually placing predicted Fab structures on the crystal structure of wild-type Prn and are not based on actual co-crystal structures or docking simulations. These models conceptually demonstrate how spatial separation or overlap of different epitopes could influence the ability of Prn to bind two Fabs simultaneously.

Conversely, in cases of antibody pairs targeting close or overlapping epitopes (2B1/1E7 and 1F2/2E9), the spectra reveal complexes consistent with Ab·Prn, Ab·[Prn]_2_, [Ab]_2_·Prn and [Ab]_2_·[Prn]_2_ (Fig. 3C and S14). For these two full-length Ab pairs, the relative abundances of the [Ab]_2_·[Prn]_2_ complexes are notably lower than observed for the other four pairs of mAbs, suggesting competitive or steric challenges upon the formation of complexes in which two full-length antibodies attempt to bind to closely located epitopes on a single Prn molecule. To illustrate this concept, Fig. 3D and S14C provide conceptual models in which AlphaFold-predicted Fab fragments are positioned on the previously reported crystal structure of Prn (1DAB), highlighting the steric hindrance that may impede dual antibody binding. These data provide valuable insights into the simultaneous interactions of antibodies targeting distinct Prn epitopes. This method could serve as a pivotal tool in understanding the binding of oligoclonal and polyclonal antibodies, which can act synergistically to achieve broad epitope coverage, offering the potential to significantly enhance bactericidal activity and prevent bacterial escape. Ultimately, this could advance the development and optimization of therapies aimed at combatting bacterial resistance more effectively.

### Thermal-induced unfolding as a measurement of complex stability

The strengths of antigen–antibody interactions and the resulting complex stabilities influence vaccine protection and guide development of future anti-pertussis. In general, the stability of these interactions can directly influence pharmacological action and therapeutic efficacy. Stable interactions ensure prolonged retention of the antibody on its target antigen, which in turn achieves better immune system activation or extended neutralization of pathogens.^62^ Variable temperature ESI-MS (vT ESI-MS) is commonly used to track thermal induced unfolding of proteins and protein complexes, allowing inference of stability.^63^ As proteins unfold in solution, more protonation sites become accessible, resulting in production of higher charge states upon ESI. Owing to this correlation, the average charge state of a protein serves as a reporter for its denaturation and unfolding. In general, the relationship between average charge state and temperature can exhibit a sigmoidal profile, from which a melting temperature (*T*_m_) can be extracted in a manner analogous to established in-solution thermal stability assays.^64^ In other cases, the dependence is more linear, as high-temperature measurements often yield incomplete unfolding transitions due to competing aggregation processes that obscure ideal melting behavior. The slope of this relationship can be used to compare the stabilities of protein complexes.^65^

First, we monitored the thermal unfolding of the individual antibodies to evaluate their inherent stabilities under controlled temperature conditions. Examples of the ESI mass spectra are shown in Fig. S15 for 1E7, 2B1, 1F2, and 2E9 at 20 °C and 60 °C, in each case showing the shift to higher charge states, indicative of protein unfolding, at the higher temperature. In particular, the abundances of the lower-charge states (+21 to +24) decreased with temperature, whereas the abundances of the higher charge states (+24 to +28) increased significantly (Fig. S15A–D). ESI mass spectra were collected for 20 temperature increments (2° per increment), and the average charge state derived from the abundances of all charge states was plotted as a function of temperature in Fig. S15E. The results for the four antibodies showed competing aggregation at elevated temperatures (typically 65–70 °C), which matches typical thermal transitions reported for IgG.^55^ Hence, we used linear fitting to compare different thermal induced unfolding for the mAbs. The charge state correlation for 2E9 yields a lower slope compared to other mAbs, although the difference compared to 1E7 was non-significant (Table 1). The trend for 2B1 gives the highest slope, which implies lower thermal stability.

**Table 1:** Slopes for the linear regression analysis in Fig. 4 and S15 showing the increase in average charge state of Ab and Ab·Prn complex as a function of solution temperature

Antibody	Slope for increase in average charge state of free antibody	(*r*^2^)	Slope for increase in average charge state of 1 : 1 mAb·Prn complex	(*r*^2^)
2E9	0.020 + 0.007	0.962	0.040 + 0.001	0.987
1E7	0.027 + 0.001	0.945	0.063 + 0.001	0.984
1F2	0.032 + 0.001	0.993	0.047 + 0.003	0.993
2B1	0.05 + 0.01	0.995	0.054 + 0.002	0.987

**Fig. 4:**
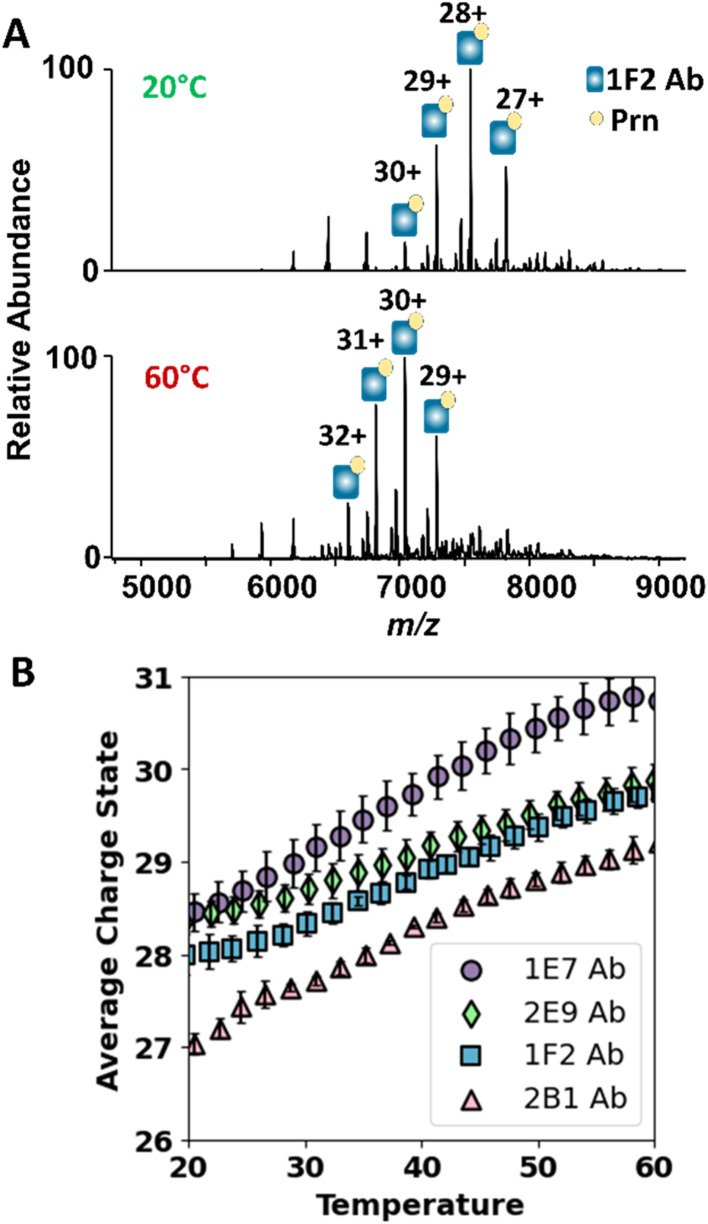
Evaluation of the thermal induced unfolding of antibody·Prn complexes using variable temperature ESI MS. (A) MS1 spectra illustrating the shift in average charge state distribution of 1F2·Prn with increasing solution temperature from 20 °C (top panel) to 60 °C (bottom panel). The spectra were obtained from solutions contain 2 µM of 1F2 and 2 µM of Prn in 250 mM ammonium acetate with 0.06% C10E5. (B) Average charge state of 1 : 1 antibody·Prn complexes as a function of solution temperature.

Next, we evaluated the thermal stabilities of the mAb·Prn complexes. ESI mass spectra for 1F2 complexes at 20 °C and 60 °C are shown in Fig. 4A and for the other complexes containing 2E9, 2B1 and 1E7 in Fig. S16. In each case, the charge states of the mAb·Prn complexes shift to higher charge states with increasing temperature, again consistent with protein unfolding. The temperature-dependent charge state trends for different mAb·Prn complexes were found to be linear, as shown in Fig. 4B, and the slopes are summarized in Table 1. The slope for the 2E9 complexes is the lowest, suggesting a modestly greater thermal stability of the 2E9·Prn complexes; however, the differences in slope among all of the antibody–Prn complexes are small and should only be interpreted qualitatively, as all antibodies bind Prn with similarly high (low-nanomolar) affinities. The ability of different mAb·Prn complexes to maintain integrity at elevated temperatures, up to 60 °C, underscores the high thermal stabilities of these mAbs, as reflected by their resistance to dissociation at high temperatures, especially 2E9. Further increase in the solution temperature up to 70 °C led to the dissociation of all the mAb·Prn complexes, except for those containing 2E9 (Fig. S17). These results support the greater stability of the complexes containing 2E9.

### Changes in antibody recognition of pertactin variants

**Fig. 5:**
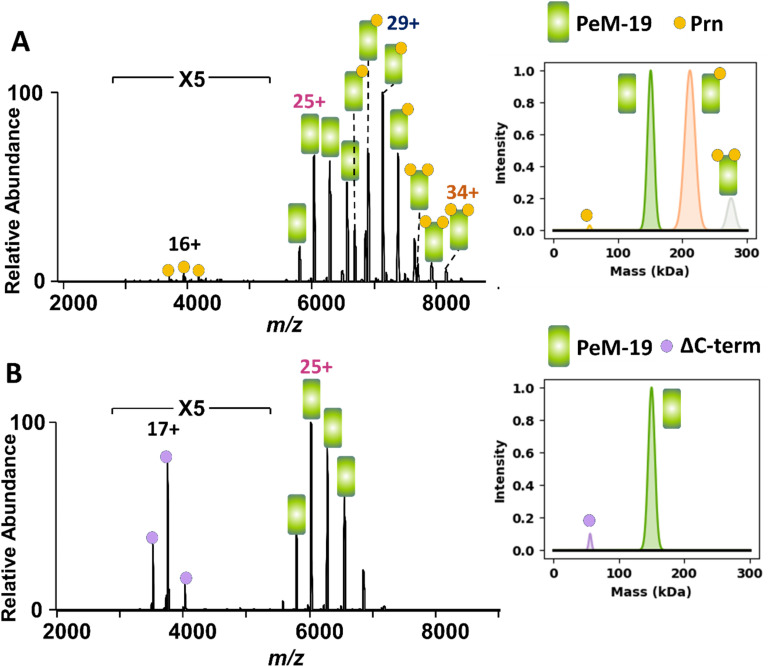
(A) MS1 spectrum obtained for a solution containing (A) 2 µM of both Prn and PeM-19 Ab or (B) 2 µM of both ΔC-term and PeM-19 Ab in 250 mM ammonium acetate with 0.06% C10E5 detergent. The spectra on the right display the Gaussian fits for the deconvoluted mass spectra.

As the 1E7, 2B1, 1F2, and 2E9 antibodies bind Prn epitopes that are well-conserved across *Bordetella* species, we extended our analysis to the loop-binding antibodies PeM-4 and PeM-19, which recognize the surface exposed variable R1 and R2 regions.^66^ These experiments were devised to assess how nMS can screen for changes in antibody binding between protein variants. Antigenic variation within the R1 and R2 repeat regions of Prn enables *B. pertussis* to evade immune responses, adding challenges to current vaccine effectiveness.^67^ Hence, we explored the use of nMS to assess the impact of R1 and R2 residue changes on antibody recognition to identify the chemical basis by which *B. pertussis* evades subsequent activation of immune responses and bacterial killing. Specifically, we evaluated the binding behavior of PeM-4 and PeM-19 to Prn variants lacking either the R1 repeat region (ΔR1; sequences in Table S2) or the C-terminal R2 repeat region (ΔC-term; sequences in Table S2). To ensure the ΔR1 Prn variant remained well-folded, the R1 loop was replaced by a glycine–serine linker [Gly_4_Ser]_2_. For the ΔC-term variant, 64 residues at the C-terminus—a section that is unstructured in the X-ray structure—were truncated (Fig. S18). These changes were designed to evaluate the specificity of these two antibodies in binding their respective Prn epitopes. PeM-4 is selective for the R1 loop,^66^ and as such, it should be effective in neutralizing wild-type Prn but not the ΔR1 Prn variant. PeM-19 is selective for the R2 loop and is expected to interact with wild-type Prn but not with the ΔC-term Prn variant. ESI mass spectra were acquired for solutions containing PeM-19 and Prn or ΔC-term Prn (each 2 µM) (Fig. 5) and solutions containing PeM-4 and Prn or ΔR1 Prn (Fig. S19).

As expected, PeM-19 formed abundant complexes with Prn and not with ΔC-term Prn (Fig. 5), and increasing the concentrations of ΔC-term Prn did not induce binding to PeM-19 (Fig. S20). Similarly, abundant PeM-4·Prn complexes are observed in Fig. S19B, but no PeM-4·ΔR1 complexes are detected in Fig. S19A, an outcome consistent with the expected binding selectivity of PeM-4. These results confirm the binding specificity of PeM-4 for the R1 loop and PeM-19 for the C-terminal R2 region of Prn, demonstrating the utility of nMS for screening antibody interactions with protein variants.

## Conclusions

Screening of specific interactions between Prn and a panel of mAbs was assessed utilizing nMS. Our findings indicated that each Fab arm in an intact antibody can bind one Prn protein for an overall 1 : 2 Ab : Prn stoichiometry, with unique greater bivalent binding through both Fab arms observed for the 2E9 mAb compared to 1F2, 2B1 and 1E7 mAbs. Furthermore, our competition experiments revealed that antibody pairs targeting distinct, non-overlapping epitopes, such as 2B1/1F2, 1E7/2E9, 2B1/2E9, and 1E7/1F2, can simultaneously bind Prn, which may be a crucial feature to mediate Fc-mediated bactericidal activities that require clustered antibody Fc domains on the bacterial surface. This may explain why these antibodies potently activate immune responses, including antibody-dependent complement cytotoxicity or phagocytosis, resulting in strong bactericidal effects. Conversely, antibodies targeting adjacent epitopes, such as the 1F2/2E9 and 2B1/1E7 pairs, produced less abundant 2 : 2 complexes consistent with competitive or steric hindrance that suppressed simultaneous binding. Native MS allowed evaluation of mAb binding specificity for different Prn variants. For instance, nMS detected that both PeM-4 and PeM-19 lost binding to their ΔR1 and ΔC-term epitopes on deletion Prn variants, highlighting the ability to implement nMS to screen different Ab candidates against various antigen mutants. Collectively, these results highlight the capability of nMS to comprehensively characterize antibody/antigen interactions and its potential to support the design of therapeutic strategies against pertussis. Future experiments will focus on incorporating charge-detection mass spectrometry^68^ for single-ion measurements that should enable the characterization of more heterogeneous antigens, even at nanomolar concentration ranges, and reveal interactions with specific proteoforms. These advancements could refine the approach, offering greater sensitivity and the ability to resolve heterogeneous samples.

## Materials and methods

### Antibody and Fab expression and purification

Antibodies binding to pertactin were purified as previously described.^59^ In brief, the antibody variable regions were cloned with mouse IgG2a heavy and light chain constant domains for IgG expression. ExpiCHO-S cells were transfected with a 1 : 3 ratio of mouse heavy chain to light chain plasmids^69^ and expressed following the manufacturer's high-titer protocol. Mouse IgG2a IgG was purified using a HiTrap^®^ Protein A column (Cytiva) on an AKTA Pure FPLC system (Cytiva). Purified protein was buffer exchanged into PBS (Amicon; Millipore Sigma) and stored at −80 °C. Protein quality was assessed with analytical size exclusion Superdex 200 (AKTA; Cytiva) and SDS-PAGE (4–20% gradient gel; Bio-Rad). Antibody identity was confirmed using native mass spectrometry after buffer exchange into 200 mM ammonium acetate (Fig. S21).

### Pertactin expression and purification

We expressed pertactin and its variants as inclusion bodies as described in ref. 59, with the following changes. Briefly, 300 mg of inclusion body was resuspended at 10 mg mL^−1^ in denaturing buffer (8 M urea, 50 mM Tris, 100 mM NaCl, 0.2 mM CaCl_2_, pH 8.0), centrifuged at 20 000 rpm for 20 min, diluted 1 : 1 in denaturing buffer, and then dialyzed against 1 L of buffer (50 mM Tris, 100 mM NaCl, 1 mM EDTA, pH 8.0) that was refreshed daily for three days. The refolded protein was centrifuged for 20 000 rpm for 20 minutes, filter-sterilized, and purified using a Strep-Tactin®XT 4Flow (IBA) gravity column according to the manufacturer's protocol. The eluate was SEC purified (Superdex 200 on an AKTA Pure FPLC system; Cytiva), concentrated to ≤2 mg mL^−1^ (Amicon; Millipore Sigma), filter-sterilized, and flash-frozen for storage at −80 °C. All pertactin produced was assessed for quality using indirect ELISA as previously described^59^ and SDS-PAGE (4–20% gradient; Bio-Rad).

### Native mass spectrometry

For nMS experiments, all recombinant proteins were buffer exchanged with 200–300 mM ammonium acetate using a gel filtration column (Bio-Spin P-40 Gel columns, BioRad) with the addition of 2× critical micelle concentration of different detergents in the case of Prn. For the screening of the interactions between the mAb panel (Table S3) and Prn antigen, 2 µM of different mAbs were incubated with equimolar concentration of Prn antigen in 250 mM ammonium acetate with 0.06% of C10E5 for 1 hour at room temperature, followed by nMS analysis. For the titration experiment, the binding interactions of 2 µM of different antibodies with increasing concentrations of Prn were measured using nMS. This experiment aimed to observe the formation of 1 : 1 and 1 : 2 antibody·Prn complexes, providing semiquantitative insights about the binding affinity and stoichiometry.

The ability of nMS to assess the specificity of the interaction between each mAb and various Prn mutants was also evaluated. This assessment is critical for verifying the binding interactions of the mAbs with Prn mutants, which may contribute to the emerging vaccine resistance. In this experiment, wild-type Prn, along with two other mutants (ΔR1 and ΔC-terminal), was assessed for binding and complex formation capabilities with equimolar concentrations of R1-specific (PeM-4) and R2-specific (PeM-19) mAbs.^66^ In addition, competition experiments were performed to investigate the simultaneous binding of different mAb pairs to Prn. Equimolar concentrations of two antibodies were incubated with an equal concentration of Prn, followed by nMS analysis. The MS1 spectra were further analyzed to track the simultaneous binding of the two antibodies to Prn.

For all these nMS experiments, 5 µL of the sample solution was loaded into in-house fabricated borosilicate capillary coated with Au/Pd for electrospray ionization using a nano ESI source on a Q Exactive Plus Ultra High Mass Range (UHMR) Orbitrap mass spectrometer (Thermo Fisher Scientific, Bremen, Germany). The MS instrument parameters were optimized to preserve noncovalent interactions and avoid undesired protein unfolding or dissociation. The optimized parameters were a spray voltage of 0.9–1.1 kV, capillary temperature of 225 °C, trapping gas pressure set to 5.1–9.0 × 10^−10^ mbar, S-lens RF level of 200% and a resolution of 1563–3125. A desolvation voltage ranging from −50 to −100 V was utilized to desolvate non-covalent complexes and effectively release proteins from detergent micelles (Fig. S2 and S3). Deconvolution of the recorded nMS spectra was performed using UniDec software, followed by Gaussian fit for simplification (Fig. S22).^70^ The following parameters were configured: the *m*/*z* range was set between 1000 and 10 000 without applying background subtraction. The charge state range was specified from 1 to 50, and the mass range was defined from 25 000 to 500 000 Da, with mass sampling at every 10 Da. Charge state distributions were smoothed, and automatic *m*/*z* peak width determination was utilized. Peak detection was configured with a range of 100 Da and a detection threshold of 10%. Other experimental details are provided in the SI.

### Biolayer interferometry

The determination of equilibrium constants (*K*_d_) of different IgGs were performed using an Octet Red96 (ForteBio) instrument. Anti-mouse IgG Fc (AMC) (ForteBio) biosensors were hydrated in a 96-well black plate containing 200 µL per well kinetic buffer (HEPES buffer with 0.01% BSA and 0.002% Tween-20) for 10 min at 24 °C. Following hydration, the biosensors were loaded with different mAbs at a concentration of 10 nM in kinetic buffer until reaching a response of 0.5 nm. The association phase was monitored for 120 seconds as the biosensors were incubated with serial dilutions of Prn, starting from an initial concentration of 50 nM, with 1 : 2 dilutions. Finally, the dissociation phase was subsequently observed by incubating the biosensors in kinetic buffer for five minutes. Global fit *K*_d_ calculations were determined using the Octet analysis software from the response values collected during the association step.

### Variable temperature (vT) ESI

Variable temperature ESI-MS experiments were performed using an in-house built ESI source that allows modulation of solution temperature during collection of ESI mass spectra.^64,65^ This in-house built setup includes an aluminum block housing to control solution temperature inside the nano ESI emitter through a three-tier Peltier thermoelectric cooler. Temperature control and monitoring were managed by a TE Technologies controller (TC-720 OEM) and a thermistor. The vT-ESI system was integrated with a standard ESI nano source for application of the spray voltage. Average charge state distributions were calculated using a Custom MATLAB script with linear regression fitting to describe thermal induced unfolding of different free mAbs and mAb·Prn complexes.

### Structure prediction of Prn and various mAb Fabs *via* AlphaFold

The folded structures of Fabs of different mAbs were predicted using AlphaFold2 *via* the publicly available AlphaFold CoLab notebook (DeepMind/EMBL-EBI).^71,72^ The predictions were performed using default settings with no template constraints. The top-ranked models, based on the model confidence scores (pLDDT), were used for subsequent visualization. The resulting structures were rendered as molecular surfaces using UCSF ChimeraX (version 1.8).^73^ To represent antibody binding regions, generic Fab fragments were manually positioned on the Prn surface based on binding data obtained from nMS and antibody competition experiments. These Fab positions are purely illustrative and were not derived from docking simulations or structural data.

## Author contributions

Conceptualization: Mohamed I. Gadallah, Virginia K. James, and Jennifer S. Brodbelt; methodology: Mohamed I. Gadallah, Kate A. McConnell, and Kelli M. Hager; writing – original draft: Mohamed I. Gadallah; writing – review and editing: Mohamed I. Gadallah, Kate A. McConnell, Kelli M. Hager, Annalee W. Nguyen, Jennifer A. Maynard, and Jennifer S. Brodbelt; supervision: Jennifer A. Maynard and Jennifer S. Brodbelt; funding acquisition: Jennifer A. Maynard and Jennifer S. Brodbelt.

## Conflicts of interest

The authors declare no competing financial interest.

## Supplementary Material

SC-OLF-D5SC09702A-s001

## Data Availability

All data have been deposited into the jPOST public repository with the accession number JPST004223. Supplementary information (SI): sequence information and mass spectra of all proteins, variable temperature ESI-MS results, equilibrium constant measurements, ESI-MS titration experiments, sequence alignment, and additional experimental details. See DOI: https://doi.org/10.1039/d5sc09702a.
