# Supplementary material for: Screening pertactin-specific antibodies and evaluating competitive epitope recognition by native mass spectrometry
Source: Chem Sci. 2026 Mar 17;17(18):9108–20. doi: 10.1039/d5sc09702a (PMC12994607; doi:10.1039/d5sc09702a)
Supplement: SC-017-D5SC09702A-s001 [file SC-017-D5SC09702A-s001.pdf]

Supplementary Information (SI) for Chemical Science.
This journal is © The Royal Society of Chemistry 2026

Supporting Information

Screening pertactin-specific antibodies and evaluating competitive epitope recognition by native mass spectrometry

Mohamed I. Gadallah[1,2], Kate A McConnell[3], Kelli Hager[3], Virginia K. James[1], , Annalee W. Nguyen[4], Jennifer A. Maynard[4], Jennifer S. Brodbelt[1]

[1]Department of Chemistry, The University of Texas at Austin, Austin, TX 78712, United States

[2]Department of Pharmaceutical Analytical Chemistry, Faculty of Pharmacy, Assuit University, Assuit 71526, Egypt

[3]Department of Molecular Biosciences, The University of Texas at Austin, Austin, TX 78712, United States

[4]Department of Chemical Engineering, The University of Texas at Austin, Austin, TX 78712, United States

Correspondence to: Jennifer S. Brodbelt, jbrodbelt@cm.utexas.edu

Content	Page no.
**Table S1.** Summary of *m/z* values, corresponding charge states, and deconvoluted masses for different antibody•Prn complexes.	S4
**Table S2.** Sequences and monoisotopic masses of wild-type Prn and different mutants. The amino acids highlighted in green represent the RGD tripeptide motif. The amino acids highlighted in blue span the R1 loop, and the amino acids highlighted red span the R2 loop.	S5
**Table S3.** Antibody variable heavy and light chain sequences with Kabat CDR definitions.	S6-S8
**Figure S1.** The crystal structures for maturation of Prn antigen (Q8RSU0) through cleavage of the extra C-terminal Porin domain. R1 loop is shaded in cyan, the RGD tripeptide motif is shaded in green, and the C-terminal R2 loop is shaded in purple.	S9
**Figure S2. Tuning of in-source trapping desolvation voltage for removal of detergents from Prn antigen.** ESI mass spectra of a solution of 5 µm Prn after buffer exchange in 300 mM ammonium acetate with 2X critical micelle concentration demonstrating the effect of desolvation voltage on the release of Prn from different detergent micelles. The results indicate that C10E5 and C8E4 are easily removed, with C10E5 having a lower critical micelle concentration and providing better spray stability. In contrast, OG and DDM require higher desolvation voltages for effective removal, with OG producing higher charge states..	S10
**Figure S3.** Heatmaps showing the mean abundances of charge states 16+ (top graph) and 15+ (bottom graph) obtained upon ESI-MS analysis of a solution of 5 µm Prn after buffer exchange in 300 mM ammonium acetate with 2X critical micelle concentration at varying desolvation voltages (10 V to 250 V) and different detergents (C10E5, C8E4, DMM, OG). Detergents C10E5 and C8E4 result in the highest Prn signals and cleanest spectra, making them the most effective for this study.	S11
**Figure S4. Expression of wild-type Prn and different mutants.** (A) SDS gel image confirming the expression of wild-type Prn and different mutants. (B) ESI-MS spectra of wild-type Prn and different mutants after buffer exchange into 300 mM ammonium acetate with 0.06% C10E5 detergent. Solutions analyzed using the UHMR mass spectrometer with 0.9-1.1 kV as spray voltage, 3 trapping gas, corresponding to a UHV gauge reading of around 2.5 × 10–10 to 3 × 10–10 mbar, and -80 V to -100 V as desolvation voltage.	S12
**Figure S5. Screening of the interaction of therapeutic mAbs targeting Prn using native MS.** MS1 spectra were obtained for solutions containing 2 µM of one antibody (A: NC, B: 2E9, C: 2B1, D: 1E7) and 2 µM of Prn in 250 mM ammonium acetate with 0.06% of C10E5. MS data was collected using positive polarity with a spray voltage of 0.9-1.1 kV, in-source trapping -100 to -150 V depending on the antibody, and 9 trapping gas, corresponding to a UHV gauge reading of around 9 × 10–10 to 9.5 × 10–10 mbar. The isotype NC antibody did not interact with Prn, as evidenced by the absence of peaks related to complex formation. In contrast, other antibodies resulted in formation of 1•1 and 1•2 mAb●Prn complexes in various charge states.	S13
**Figure S6: Application of the SLOMO method for studying the interaction of Prn and 1E7 mAb.** Representative ESI mass spectra acquired at different times after mixing solution 1 (0.1 µM Prn + 1 µM 1E7 mAb) and solution 2 (1 µM Prn + 1 µM 1E7 mAb). The slow diffusion and lack of full equilibration, likely due to the large molecular sizes of the interaction partners, demonstrate the impracticality of this method for full length antibody–antigen systems.	S14

Content	Page no.
**Figure S7. Equilibrium constant (K_d) measurements using biolayer interferometry for different mAbs targeting Prn.** Data collected using anti-mice Fc BLI biosensors coated with 10 nM of mAbs. Then each biosensor was dipped into six serial dilutions of Prn, starting from an initial concentration of 50 nM, with 1:2 dilutions (corresponding to Prn concentrations of 50 nM, 25 nM, 12.5 nM, 6.25 nM, 3.125 nM, and 1.56 nM) to allow association followed by incubating the biosensors in kinetic buffer for five minutes to monitor the dissociation phase. The start of the dissociation phase is marked by a vertical red line in each graph. Equilibrium constants of less than 1nM were determined from the recorded response using 1:1 binding model with Octet analysis software using single replicate.	S15
**Figure S8. Titration experiment of 2E9 with Prn using native MS**. (A) ESI mass spectra obtained after incubating various concentrations of Prn (0.5, 1, 2, or 4 µM Prn) with 2 µM 2E9 antibody in 250 mM ammonium acetate with 0.06% of C10E5. The corresponding deconvoluted spectra are shown on the right. The spectra display an increase in relative abundances of both 2E9•Prn and 2E9•$[Prn]_2$ complexes as the Prn concentration increases. (B) Bar graph showing the relative intensities of the 2E9•Prn and 2E9•$[Prn]_2$ complexes at different Prn concentrations. (C) MS1 spectrum of a solution containing 8 uM Prn with 2 uM of 2E9 antibody, confirming that a further increase of Prn concentration does not lead to the formation of higher stoichiometry complexes.	S16
**Figure S9. Titration experiment of 1F2 using native MS**. (A) ESI mass spectra obtained after incubating various concentrations of Prn (0.5, 1, 2, or 4 µM Prn) with 2 µM 1F2 antibody in 250 mM ammonium acetate with 0.06% of C10E5. The corresponding deconvoluted spectra are shown on the right. The spectra display an increase in relative abundances of both 1F2•Prn and 1F2•$[Prn]_2$ complexes as the Prn concentration increases. (B) Bar graph showing the relative intensities of the 1F2•Prn and 1F2•$[Prn]_2$ complexes at different Prn concentrations. (C) MS1 spectrum of a solution containing 8 uM Prn with 2 uM of 1F2 antibody confirming that a further increase of Prn concentration does not lead to the formation of higher stoichiometry complexes.	S17
**Figure S10. Titration experiment of 2B1 using native MS**. (A) ESI mass spectra obtained after incubating various concentrations of Prn (0.5, 1, 2, or 4 µM Prn) with 2 µM 2B1 antibody in 250 mM ammonium acetate with 0.06% of C10E5. The corresponding deconvoluted spectra are shown on the right. The spectra display an increase in relative abundances for both 2B1•Prn and 2B1•$[Prn]_2$ complexes as the Prn concentration increases. (B) Bar graph showing the relative intensities of the 2B1•Prn and 2B1•$[Prn]_2$ complexes at different Prn concentrations. (C) MS1 spectrum of a solution containing 8 uM Prn with 2 uM of 2B1 antibody confirming that a further increase of Prn concentration does not lead to the formation of higher stoichiometry complexes.	S18
**Figure S11. Titration experiment of 1E7 using native MS**. (A) ESI mass spectra obtained after incubating various concentrations of Prn (0.5, 1, 2, or 4 µM Prn) with 2 µM 1E7 antibody in 250 mM ammonium acetate with 0.06% of C10E5. The corresponding deconvoluted spectra are shown on the right. The spectra display an increase in relative abundances for both 1E7•Prn and 1E7•$[Prn]_2$ complexes as the Prn concentration increases. (B) Bar graph showing the relative intensities of the 1E7•Prn and 1E7•$[Prn]_2$ complexes at different Prn concentrations. (C) MS1 spectrum of a solution containing 8 uM Prn with 2 uM of 1E7 antibody confirming that a further increase of Prn concentration does not lead to the formation of higher stoichiometry complexes.	S19
**Figure S12. Comparison of antibody–antigen complex stoichiometries determined by native MS.** Gaussian fits to deconvoluted mass spectra obtained from ESI-MS of 2 µM antibody solutions incubated with 4 µM Prn in 250 mM ammonium acetate containing 0.06 % C10E5 detergent. (A) 1F2 forms both 1:1 and 1:2 mAb•Prn complexes, whereas (B) 2E9 predominantly yields the 1:2 mAb•$[Prn]_2$ complex. The results illustrate distinct stoichiometric distributions among antibodies, with 2E9 exhibiting a higher fraction of the bivalent complex under these conditions.	S20
**Figure S13. Screening of simultaneous binding of different mAb pairs targeting different Prn epitopes. (A-C)** MS1 spectra obtained from incubation of 5 µM 1E7/2E9, 2B1/2E9 and 1E7/1F2, respectively, with equimolar concentration of Prn. Graphs on the right show Gaussian fits to the deconvoluted mass spectra. (D-F) Schematic representation of Prn+2E9-Fab+1E7-Fab, Prn+2B1-Fab+2E9-Fab and Prn+1E7- Fab+1F2-Fab, respectively, with AlphaFold-predicted structure Prn shaded gray (1DAB), 2E9-Fab shaded green, 2B1-Fab shaded pink, 1F2-Fab shaded blue and 1E7-Fab shaded purple, all shown as molecular surfaces. The presence of ion peaks corresponding to complexes containing Prn and two Abs indicates that Abs that bind to distant epitopes can simultaneously bind to the Prn antigen.	S21

Content	Page no.
**Figure S14. Screening of simultaneous binding of different mAb pairs targeting different Prn epitopes. (A)** MS1 spectrum obtained from incubation of 5 μM 1F2/2E9 with equimolar concentration of Prn. B) Graph shows Gaussian fits to deconvoluted mass spectrum. (C) Schematic representation of Prn+1F2-Fab+2E9-Fab with AlphaFold-predicted structure Prn shaded gray (1DAB), 2E9-Fab shaded green, and 1F2-Fab shaded blue, all shown as molecular surfaces. Simultaneous binding of 2E9 and 1F2 is suppressed, resulting in low intensity peaks for the $[Ab]_2$●Prn complexes.	S22
**Figure S15. Evaluation of the thermal stability of different antibodies using vT-ESI MS.** (A,B,C,D) MS1 spectra illustrating the shift in average charge state distribution with increasing solution temperature, ranging from 20°C (top panels) to 60 °C (bottom panels). The spectra were obtained from solutions contain 2 μM of antibody: A: 2E9 green, B; 2B1 pink, C: 1F2 blue and D: 1E7 purple. (E) Plot of the average charge state of each antibody as a function of solution temperature.	S23
**Figure S16. Evaluation of the thermal induced unfolding of different antibody•Prn complexes using vT-ESI native MS.** (A,B,C) MS1 spectra illustrating the shift in average charge state distribution with increasing solution temperature, ranging from 20°C (top panels) to 60 °C (bottom panels). The spectra were obtained for solutions containing 2 μM of an antibody (A; 2E9 green, B: 2B1 pink, C: 1E7 purple) with 2 μM of Prn in 250 mM ammonium acetate with 0.06% C10E5.	S24
**Figure S17. Utilization of vT-ESI for evaluation of thermal stability of different mAb•Prn complexes at elevated solution temperature of 70°C.** MS1 spectra demonstrate the thermal induced dissociation of different mAb•Prn complexes at high solution temperature. Notably, 2E9 shows remarkable stability; its complex remains intact and does not release the Prn antigen even at 70°C.	S25
**Figure S18.** Sequence alignment of Prn, ΔR1 and ΔC-term mutants from *Bordetella Pertussis* generated with ClustalOmega	S26
**Figure S19**. (A) MS1 spectrum obtained for a solution containing 2 μM of both ΔR1 and PeM-4 Ab in 250 mM ammonium acetate with 0.06% C10E5 detergent (B) MS1 spectrum obtained for a solution containing 2 μM of both Prn and PeM-4 Ab in 250 mM ammonium acetate with 0.06% C10E5 detergent. The insets highlight the Gaussian fits for the deconvoluted mass spectra.	S27
**Figure S20. Monitoring interactions of high concentrations of ΔR1** and ΔC-term **mutants with specific monoclonal antibodies.** (A) Mass spectra of solutions containing 4 μM ΔR1 mutant incubated with 2 μM R1-specific monoclonal antibody PeM-4, and (B) 4 μM ΔC-term mutant incubated with 2 μM C-term-specific monoclonal antibody PeM-19. The experiments demonstrate the absence of binding interactions even at twice the protein concentration.	S28
**Figure S21. ESI MS1 spectra of monoclonal antibodies targeting different Prn epitopes**. Each antibody was buffer exchanged into 200 mM ammonium acetate and analyzed using the UHMR mass spectrometer with 1.0 kV to 1.1 kV as spray voltage, 6 trapping gas and -100V to -120V as desolvation voltage.	S29
**Figure S22. Analysis of deconvoluted mass spectra obtained from UniDec[2] using Gaussian fitting and simplification**. Left panel represent deconvoluted MS1 spectrum obtained from UniDec. The middle panel refines this data further using Gaussian fitting, highlighting significant peaks that correspond to different protein or protein complexes. The right panel represents a simplified version of the spectrum, where only the most prominent peaks are retained for focused analysis of the key molecular components.	S30
Antibody and Fab expression, purification, and quality control.<br>Pertactin expression, purification, and quality control	S31
References	S32

Table S1. Summary of *m/z* values, corresponding charge states, and deconvoluted masses for different antibody•Prn complexes.

Complex	*m/z*	Charge state	Deconvoluted mass (kDa)
2E9•Prn	7270	29	210.8
	7029	30	
	6799	31	
1F2•Prn	7283	29	211.6
	7040	30	
	6812	31	
2B1•Prn	7312	29	212.0
	7068	30	
	6840	31	
1E7•Prn	7309	29	211.9
	7065	30	
	6837	31	

Table S2. Sequences and monoisotopic masses of wild-type Prn and different mutants. The amino acids highlighted in green represent the RGD tripeptide motif. The amino acids highlighted in blue span the R1 loop, and the amino acids highlighted in red span the R2 loop.

Protein	Sequence	Monoisotopic mass (Da)
**Mature Pertactin (Prn)**	MGSSWSHPQFEKSSGASDWNNQSIVKTGERQHGIHIQGSDPGGVRTASGTTIKVSG<br>RQAQGILLENPAAELQFRNGSVTSSGQLSDDGIRRFLGTVTVKAGKLVADHATLANVG<br>DTWDDDGIALYVAGEQAQASIADSTLQGAGGVQIERGANVTVQRSAIVDGGLHIGAL<br>QSLQPEDLPPSRVVLRDTNVTAVPASGAPAAVSVLGASELTLDGGHITGGRAAGVAAM<br>QGAVVHLQRATIRRGDAPAGGGVPGGAVPGGAVPGGFGPGGFGPVLDGWYGVDV<br>SGSSVELAQSIVEAPELGAAIRVGRGARVTVSGGSLFAPHGNVIETGGARRFAPQAAPL<br>SITLQAGAHAQGKALLYRVLPEPVKLTLTGGADAQGDIVATELPSIPGTSIGPLDVALASQ<br>ARWTGATRAVDSLSIDNATWVMTDNSNVGALRLASDGSVDFQQPAEAGRFKVLTVN<br>TLAGSGLFRMNVFADLGLSDKLVVMQDASGQHRLWVRNSGSEPASANTLLLVQTPRG<br>SAATFTLANKDGKVDIGTYRYRLAANGNGQWSLVGAKAPPAPKPAPQPGPQPPQPQ<br>PEAPAPQPPAGRELSAAANAAVNTGGVGLASTLWYAESNALSKRLGEL	62,770
**ΔR1**	MGSSWSHPQFEKSSGASDWNNQSIVKTGERQHGIHIQGSDPGGVRTASGTTIKVSG<br>RQAQGILLENPAAELQFRNGSVTSSGQLSDDGIRRFLGTVTVKAGKLVADHATLANVG<br>DTWDDDGIALYVAGEQAQASIADSTLQGAGGVQIERGANVTVQRSAIVDGGLHIGAL<br>QSLQPEDLPPSRVVLRDTNVTAVPASGAPAAVSVLGASELTLDGGHITGGRAAGVAAM<br>QGAVVHLQRATIRRGDAPAGGGGSGGGGSVLDGWYGVDVSGSSVELAQSIVEAPEL<br>GAAIRVGRGARVTVSGGSLSAPHGNVIETGGARRFAPQAAPLSITLQAGAHAQGKALL<br>YRVLPEPVKLTLTGGADAQGDIVATELPSIPGTSIGPLDVALASQARWTGATRAVDSLSI<br>DNATWVMTDNSNVGALRLASDGSVDFQQPAEAGRFKVLTVNTLAGSGLFRMNVFA<br>DLGLSDKLVVMQDASGQHRLWVRNSGSEPASANTLLLVQTPLGSAATFTLANKDGKV<br>DIGTYRYRLAANGNGQWSLVGAKAPPAPKPAPQPGPQPPQPPQPQPEAPAPQPPAG<br>RELSAAANAAVNTGGVGLASTLWYAESNALSKRLGEL	61,659
**ΔC-term**	MGSSWSHPQFEKSSGASDWNNQSIVKTGERQHGIHIQGSDPGGVRTASGTTIKVSG<br>RQAQGILLENPAAELQFRNGSVTSSGQLSDDGIRRFLGTVTVKAGKLVADHATLANVG<br>DTWDDDGIALYVAGEQAQASIADSTLQGAGGVQIERGANVTVQRSAIVDGGLHIGAL<br>QSLQPEDLPPSRVVLRDTNVTAVPASGAPAAVSVLGASELTLDGGHITGGRAAGVAAM<br>QGAVVHLQRATIRRGDAPAGGAVPGGAVPGGAVPGGFGPGGFGPVLDGWYGVDVS<br>GSSVELAQSIVEAPELGAAIRVGRGARVTVSGGSLSAPHGNVIETGGARRFAPQAAPLS<br>ITLQAGAHAQGKALLYRVLPEPVKLTLTGGADAQGDIVATELPSIPGTSIGPLDVALASQ<br>ARWTGATRAVDSLSIDNATWVMTDNSNVGALRLASDGSVDFQQPAEAGRFKVLTVN<br>TLAGSGLFRMNVFADLGLSDKLVVMQDASGQHRLWVRNSGSEPASANTLLLVQTPLG<br>SAATFTLANKDGKVDIGTYRYRLAANGNGQWSLVGAKAPP	56,311

Table S3. Antibody variable heavy and light chain sequences with Kabat CDR definitions.

Antibody subunit: Theoretical average mass (Da)	Amino Acid Sequence	Average Deconvoluted mass (Da)
1F2 HC: 49038	EVQLVESGGGLVKPGESLKLSCAASGFTFNSYTMSWVRQTPEKRLEWVATISSGGSYTYYPDSVKGRFTISRDNAKNTLYLQMSSLKSEDTAMYYCIRVGGLFDYWGQGTTLTVSRSTAPSVYPLAPVCGDTTGSSVTLGCLVKGYFPEPVTLTWNSGSLSSGVHTFPAVLQSDLYTLSSSVTVTSSTWPSQSITCNVAHPASSTKVDKKIEPRGPTIKPCPPCKCPAPNLLGGPSVFIFPPKIKDVLMISLSPIVTCVVVDVSEDDPDVQISWFVNNVEVHTAQTQTHREDYNSTLRVVSALPIQHQDWMSGKEFKCKVNNKDLPAPIERTISKPKGSVRAPQVYVLPPPEEEMTKKQVTLTCMVTDFMPEDIYVEWTNNGKTELNYKNTEPVLDSDGSYFMYSKLRVEKKNWVERNSYSCSVVHEGLHNHHTTKSFSRTPGK	148,317
1F2 LC: 23713	(D)IQMIQSHKFMSTSVGDRVSITCKASQDVSTAVAWYQQKPGQSPKLLIYWASTRHTGVPDRFTGSGSGTDFTLTISGVQAEDLALYYCQQHYTTPWTFGGGTKLEIKRTADAAPTVSIFPPSSEQLTSGGASVVCFLNNFYPKDINVKWKIDGSERQNGVLNSWTDQDSKDSTYSMSSTLTLTKDEYERHNSYTCEATHKTSTSPIVKSFNRNEC	
2B1 HC: 49485	QVQLQQSGPELVKPGASVKISCKASGYSFTDYNMYWVKQSHGKSLEWIGYFDPYNGGIYYNQNFKGRATLTVDKSSSTAFMHLNSLTSEDSAVYYCARGGGWFRGFTYWGQGTLVTVSRSTAPSVYPLAPVCGDTTGSSVTLGCLVKGYFPEPVTLTWNSGSLSSGVHTFPAVLQSDLYTLSSSVTVTSSTWPSQSITCNVAHPASSTKVDKKIEPRGPTIKPCPPCKCPAPNLLGGPSVFIFPPKIKDVLMISLSPIVTCVVVDVSEDDPDVQISWFVNNVEVHTAQTQTHREDYNSTLRVVSALPIQHQDWMSGKEFKCKVNNKDLPAPIERTISKPKGSVRAPQVYVLPPPEEEMTKKQVTLTCMVTDFMPEDIYVEWTNNGKTELNYKNTEPVLDSDGSYFMYSKLRVEKKNWVERNSYSCSVVHEGLHNHHTTKSFSRTPGK	149,064
2B1 LC: 23663	(D)IQMTQSTSSLSASLGDRVTISCRASQDISNYLNWYQQKPDGTLNLLIYYTSRLHSGVPSRFSGSGSGTDYSLTISNLEQEDIATYFCQQGNTLPYTFGGGTKLEIKRTADAAPTVSIFPPSSEQLTSGGASVVCFLNNFYPKDINVKWKIDGSERQNGVLNSWTDQDSKDSTYSMSSTLTLTKDEYERHNSYTCEATHKTSTSPIVKSFNRNEC	
2E9 HC: 49078	QVQLQQSGPELVKPGASVKMSCKASGYTFTSNYIHWMKQRPGQGLEWIGWIYPGDGSSKYNEKFKGKTTLTADRSSSTAYMLLSSLTSEDSAIYFCANYFYAMDQWGQGTSVTVSRSTAPSVYPLAPVCGDTTGSSVTLGCLVKGYFPEPVTLTWNSGSLSSGVHTFPAVLQSDLYTLSSSVTVTSSTWPSQSITCNVAHPASSTKVDKKIEPRGPTIKPCPPCKCPAPNLLGGPSVFIFPPKIKDVLMISLSPIVTCVVVDVSEDDPDVQISWFVNNVEVHTAQTQTHREDYNSTLRVVSALPIQHQDWMSGKEFKCKVNNKDLPAPIERTISKPKGSVRAPQVYVLPPPEEEMTKKQVTLTCMVTDFMPEDIYVEWTNNGKTELNYKNTEPVLDSDGSYFMYSKLRVEKKNWVERNSYSCSVVHEGLHNHHTTKSFSRTPGK	147,603
2E9 LC: 23686	(D)IVLTQSHKFMSTSVGDRVSITCKASQDVSTAVAWYQQKPGQSPKLLIYWASTRHTGVPDRFTGSGSGTDYTLTISSVQAEDLALYYCQQHYSTPWTFGGGTKLEIKRTADAAPTVSIFPPSSEQLTSGGASVVCFLNNFYPKDINVKWKIDGSERQNGVLNSWTDQDSKDSTYSMSSTLTLTKDEYERHNSYTCEATHKTSTSPIVKSFNRNEC*	

Table S3 (continued). Antibody variable heavy and light chain sequences with Kabat CDR definitions.

Antibody subunit: Theoretical average mass (Da)	Amino Acid Sequence	Average Deconvoluted mass (Da)
1E7 HC: 49485.99	QVQLQQPAAELARPGASVKMSCRASGYTFTTYTMHWVKQRPGQGLEWI<br>GYINPSSGYTDYNQKFRDKITLTADKSSSTAYMQLSSLTSGDSAVYYCARED<br>YYGQGFTYWGQGTLVTVSRSTAPSVYPLAPVCGDTTGSSVTLGCLVKGYFP<br>EPVTLTWNSGSLSSGVHTFPAVLQSDLYTLSSSVTVTSSTWPSQSITCNVAH<br>PASSTKVDKKIEPRGPTIKPCPPCKCPAPNLLGGPSVFIFPPKIKDVLMISLSPI<br>VTCVVVDVSEDDPDVQISWFVNNVEVHTAQTQTHREDYNSTLRVVSALPI<br>QHQDWMSGKEFKCKVNNKDLPAPIERTISKPKGSVRAPQVYVLPPPEEEM<br>TKKQVTLTCMVTDFMPEDIYVEWTNNGKTELNYKNTEPVLDSDGSYFMY<br>SKLRVEKKNWVERNSYSCSVVHEGLHNHHTTKSFSRTPGK*	149,106
1E7 LC: 23721.22	(D)IVMTQSTSSLSASLGDRVTISCRASQDISNYLNWYQQKPDGTVKLLIYYT<br>SRLHSGVPSRFSGSGSGTDYSLTISNLEQEDIATYFCQQGNMFPWTFGGGT<br>KLELKRTADAAPTVSIFPPSSEQLTSGGASVVCFLNNFYPKDINVKWKIDGS<br>ERQNGVLNSWTDQDSKDSTYSMSSTLTLTKDEYERHNSYTCEATHKTSTSP<br>IVKSFNRNEC*	
PeM-4 HC: 49297.84	EVQLVESGGGLVKPGGSLKLSCAASGFTFSDYYMYWVRQTPEKRLEWVAT<br>ISDGGTYTSYPDSVKGRFTISRDNAKNNLYLQMSSLKSEDTAMYYCVRWLG<br>DAVDYWGQGTSVTVSSRSTAPSVYPLAPVCGDTTGSSVTLGCLVKGYFPEP<br>VTLTWNSGSLSSGVHTFPAVLQSDLYTLSSSVTVTSSTWPSQSITCNVAHP<br>ASSTKVDKKIEPRGPTIKPCPPCKCPAPNLLGGPSVFIFPPKIKDVLMISLSPIV<br>TCVVVDVSEDDPDVQISWFVNNVEVHTAQTQTHREDYNSTLRVVSALPIQ<br>HQDWMSGKEFKCKVNNKDLPAPIERTISKPKGSVRAPQVYVLPPPEEEMT<br>KKQVTLTCMVTDFMPEDIYVEWTNNGKTELNYKNTEPVLDSDGSYFMYS<br>KLRVEKKNWVERNSYSCSVVHEGLHNHHTTKSFSRTPGK*	149,950
PeM-4 LC: 24268.18	DIVMTQAPLTLSVTFGQPASISCKSSQSLLYSNGKTYLNWLLQRPGQSPKRL<br>IYLVSKLDSGVPDRFTGSGSGTDFTLKINRVETEDLGVYYCVQGTHFPLTFG<br>AGTKLELKRTADAAPTVSIFPPSSEQLTSGGASVVCFLNNFYPKDINVKWKI<br>DGSERQNGVLNSWTDQDSKDSTYSMSSTLTLTKDEYERHNSYTCEATHKT<br>STSPIVKSFNRNEC*	

Table S3 (continued). Antibody variable heavy and light chain sequences with Kabat CDR definitions.

Antibody subunit: Theoretical average mass (Da)	Amino Acid Sequence	Average Deconvoluted mass (Da)
PeM-19 HC: 49223.74	EVQLQQSGPELVKPGASVKIPCKASGYTFTDYNMDWVKQSHGKSLEWIGDINPNT<br>GETIYNQKFEGKASLTVDKSSNTVYMELRSLTSEDTAVYYCARVPFYFDYWGQGTT<br>LTVSRSTAPSVYPLAPVCGDTTGSSVTLGCLVKGYFPEPVTLTWNSGSLSSGVHTFP<br>AVLQSDLYTLSSSVTVTSSTWPSQSITCNVAHPASSTKVDKKIEPRGPTIKPCPPCKC<br>PAPNLLGGPSVFIFPPKIKDVLMISLSPIVTCVVVDVSEDDPDVQISWFVNNVEVHT<br>AQTQTHREDYNSTLRVVSALPIQHQDWMSGKEFKCKVNNKDLPAPIERTISKPKGS<br>VRAPQVYVLPPPEEEMTKKQVTLTCMVTDFMPEDIYVEWTNNGKTELNYKNTEP<br>VLDSDGSYFMYSKLRVEKKNWVERNSYSCSVVHEGLHNHHTTKSFSRTPGK*	150,860
PeM-19 LC: 24587.36	DIVMSQSPSSLTVSVGEKVTMSCKSSQSLLYSRDQKNYLAWYQQKPGQSPKMLIH<br>WASTRASGVPDRFTGSGSGTDFTLTISSVKAEDLAVYYCQQYYFYPRTFGGGTKLEL<br>KRTADAAPTVSIFPPSSEQLTSGGASVVCFLNNFYPKDINVKWKIDGSERQNGVLN<br>SWTDQDSKDSTYSMSSTLTLTKDEYERHNSYTCEATHKTSTSPIVKSFNRNEC*	
Chimeric human 2E9 Fab HC: 23350.28	QVQLQQSGPELVKPGASVKMSCKASGYTFTSNYIHWMKQRPGQGLEWIGWIYP<br>GDGSSKYNEKFKGKTTLTADRSSSTAYMLLSSLTSEDSAIYFCANYFYAMDQWGQG<br>TSVTVSASTKGPSVFPLAPSSKSTSGGTAALGCLVKDYFPEPVTVSWNSGALTSGVH<br>TFPAVLQSSGLYSLSSVVTVPSSSLGTQTYICNVNHKPSNTKVDKRVEPKSC*	
Chimeric human 2E9 Fab LC: 23416.12	(D)IVLTQSHKFMSTSVGDRVSITCKASQDVSTAVAWYQQKPGQSPKLLIYWASTR<br>HTGVPDRFTGSGSGTDYTLTISSVQAEDLALYYCQQHYSTPWTFGGGTKLEIKRTV<br>AAPSVFIFPPSDEQLKSGTASVVCLLNNFYPREAKVQWKVDNALQSGNSQESVTE<br>QDSKDSTYSLSSTLTLSKADYEKHKVYACEVTHQGLSSPVTKSFNRGEC*	
Chimeric human 1E7 Fab HC: 23757.61	QVQLQQPAAELARPGASVKMSCRASGYTFTTYTMHWVKQRPGQGLEWIGYINP<br>SSGYTDYNQKFRDKITLTADKSSSTAYMQLSSLTSGDSAVYYCAREDYYGQGFTYW<br>GQGTLVTVSASTKGPSVFPLAPSSKSTSGGTAALGCLVKDYFPEPVTVSWNSGALT<br>SGVHTFPAVLQSSGLYSLSSVVTVPSSSLGTQTYICNVNHKPSNTKVDKRVEPKSC*	
Chimeric human 1E7 Fab LC: 23451.09	(D)IVMTQSTSSLSASLGDRVTISCRASQDISNYLNWYQQKPDGTVKLLIYYTSRLHS<br>GVPSRFSGSGSGTDYSLTISNLEQEDIATYFCQQGNMFPWTFGGGTKLELKRTVAA<br>PSVFIFPPSDEQLKSGTASVVCLLNNFYPREAKVQWKVDNALQSGNSQESVTEQDS<br>KDSTYSLSSTLTLSKADYEKHKVYACEVTHQGLSSPVTKSFNRGEC*	

Note 1: all variable region light chains should start with a D residue; due to a cloning artifact this was omitted from some antibodies and is accordingly indicated as a (D).

Note 2: Only one predicted N-linked glycosylation site (N-X-S/T motif), highlighted in yellow on 1E7 heavy chain, predicted using the NetNGlyc 1.0 Server.

NetNGlyc 1.0 Server (DTU Bioinformatics; https://services.healthtech.dtu.dk/services/NetNGlyc-1.0/).1

Note 3: Molecular weights of individual antibody chains were predicted using the Expasy ProtParam Server. 1E7 HC predicted molecular weights do not include predicted N-linked glycosylation sites.

Expasy ProtParam Server (Gasteiger E. *et al.*, https://web.expasy.org/protparam/)

Note 4: Complementarity-determining regions (CDRs) are underlined.

Figure S1. The crystal structures for maturation of Prn antigen (1DAB) through cleavage of the extra C-terminal Porin domain. R1 loop is shaded in cyan, the RGD tripeptide motif is shaded in green, and the C-terminal R2 loop is shaded in purple.

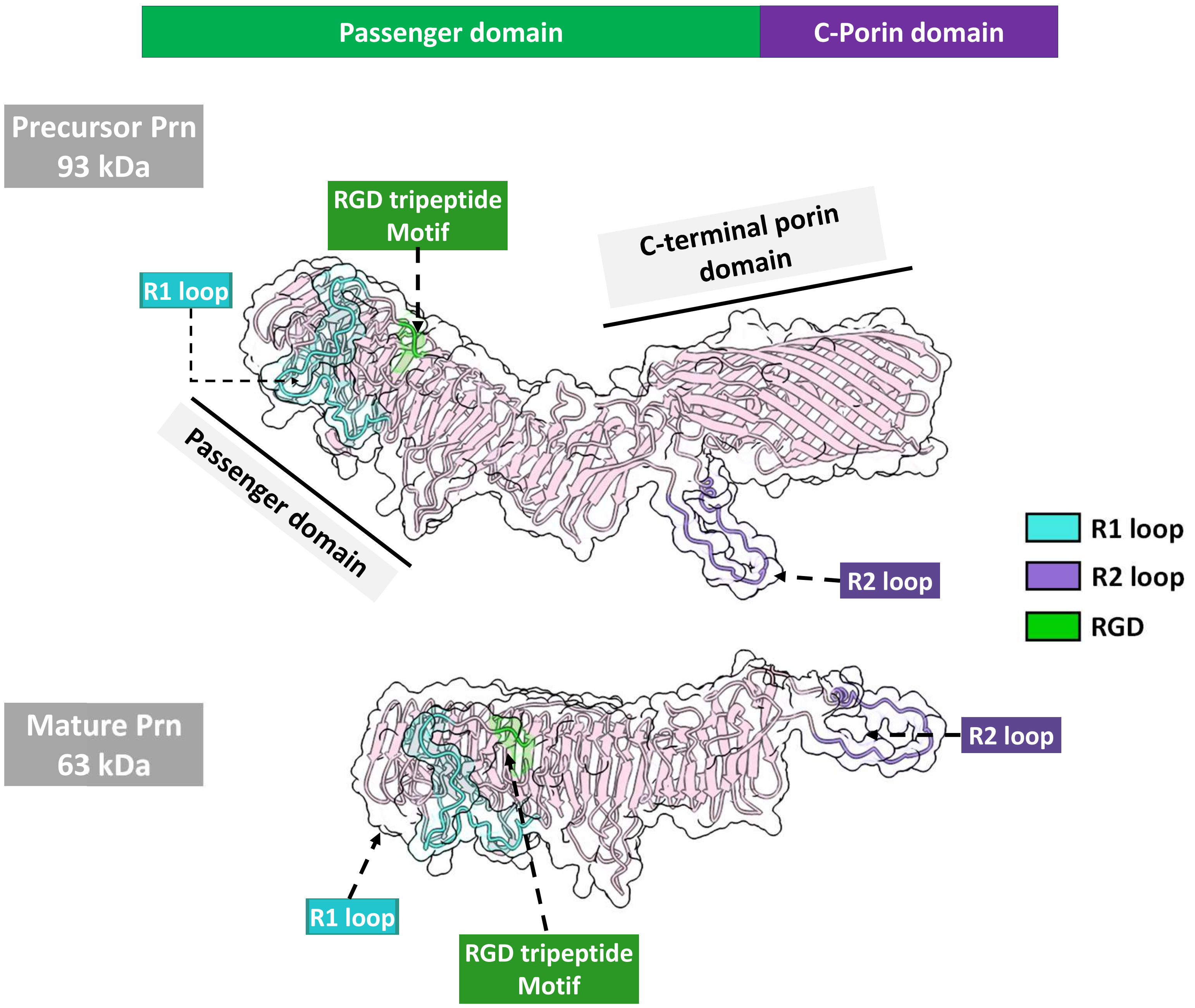

Figure S2. Tuning of in-source trapping desolvation voltage for removal of detergents from Prn antigen. ESI mass spectra of a solution of 5 μm Prn after buffer exchange in 300 mM ammonium acetate with 2X critical micelle concentration demonstrating the effect of desolvation voltage on the release of Prn from different detergent micelles. The results indicate that C10E5 and C8E4 are easily removed, with C10E5 having a lower critical micelle concentration and providing better spray stability. In contrast, OG and DDM require higher desolvation voltages for effective removal, with OG producing higher charge states.

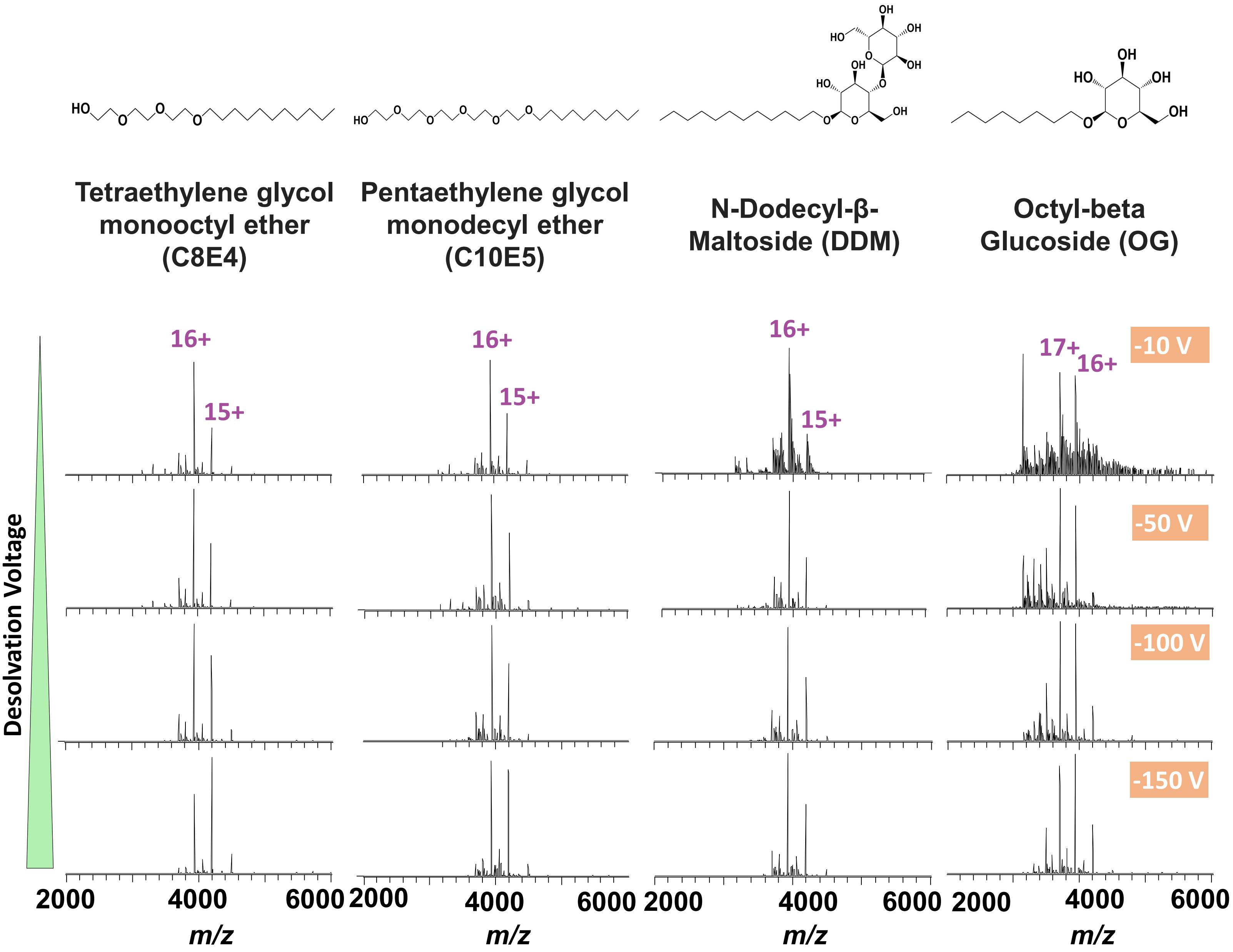

Figure S3. Heat maps showing the mean abundances of charge states 16+ (top graph) and 15+ (bottom graph) obtained upon ESI-MS analysis of a solution of 5 μm Prn after buffer exchange in 300 mM ammonium acetate with 2X critical micelle concentration at varying desolvation voltages (10 V to 250 V) and different detergents (C10E5, C8E4, DMM, OG). Detergents C10E5 and C8E4 result in the highest Prn signals and cleanest spectra, making them the most effective for this study.

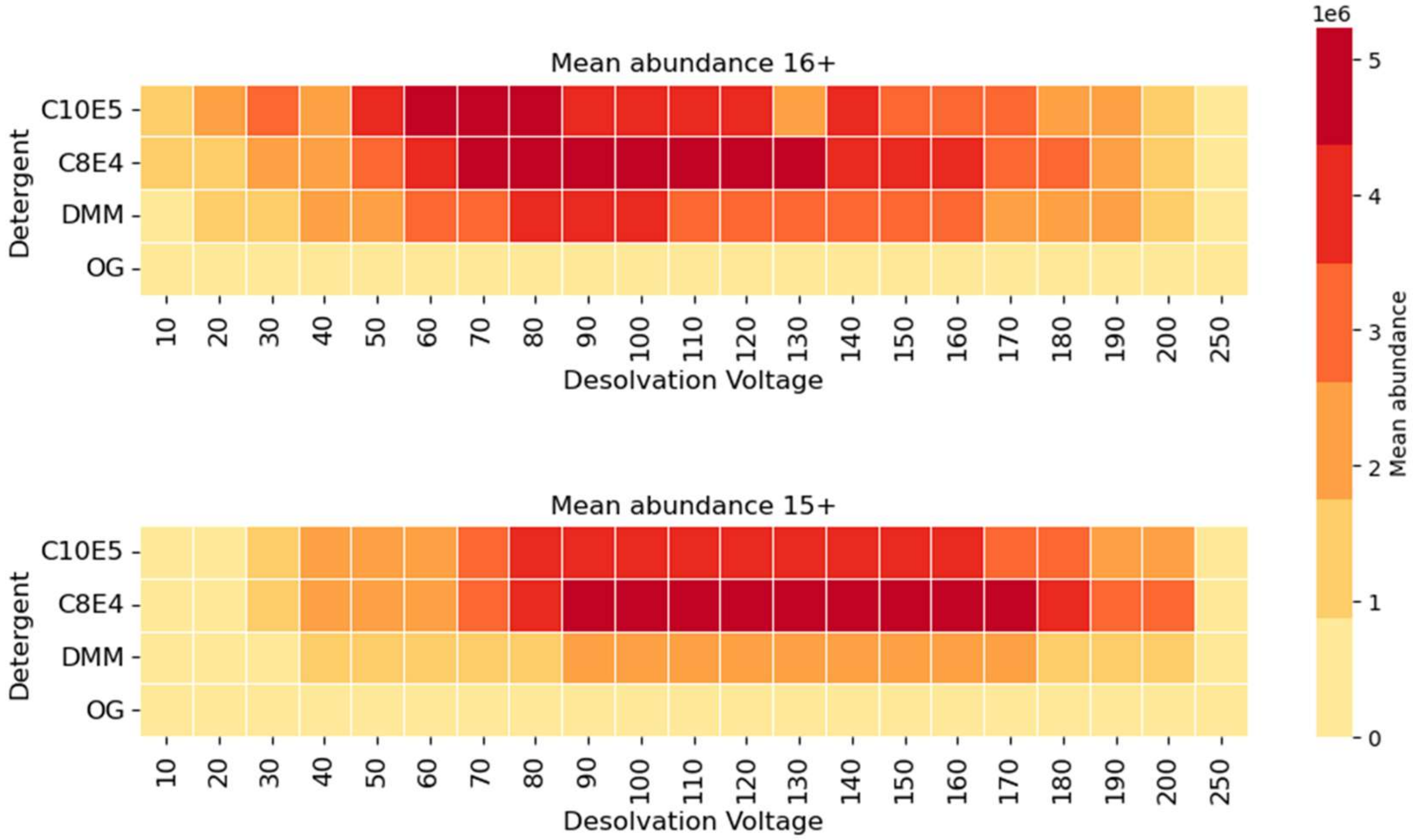

Figure S4. Expression of wild-type Prn and different mutants. (A) SDS gel image confirming the expression of wild-type Prn and different mutants. (B) ESI-MS spectra of wild-type Prn and different mutants after buffer exchange into 300 mM ammonium acetate with 0.06% C10E5 detergent. Solutions analyzed using the UHMR mass spectrometer with 0.9-1.1 kV as spray voltage, 3 trapping gas, corresponding to a UHV gauge reading of around 2.5 × 10^{-10} to 3 × 10^{-10} mbar, and -80 V to -100 V as desolvation voltage.

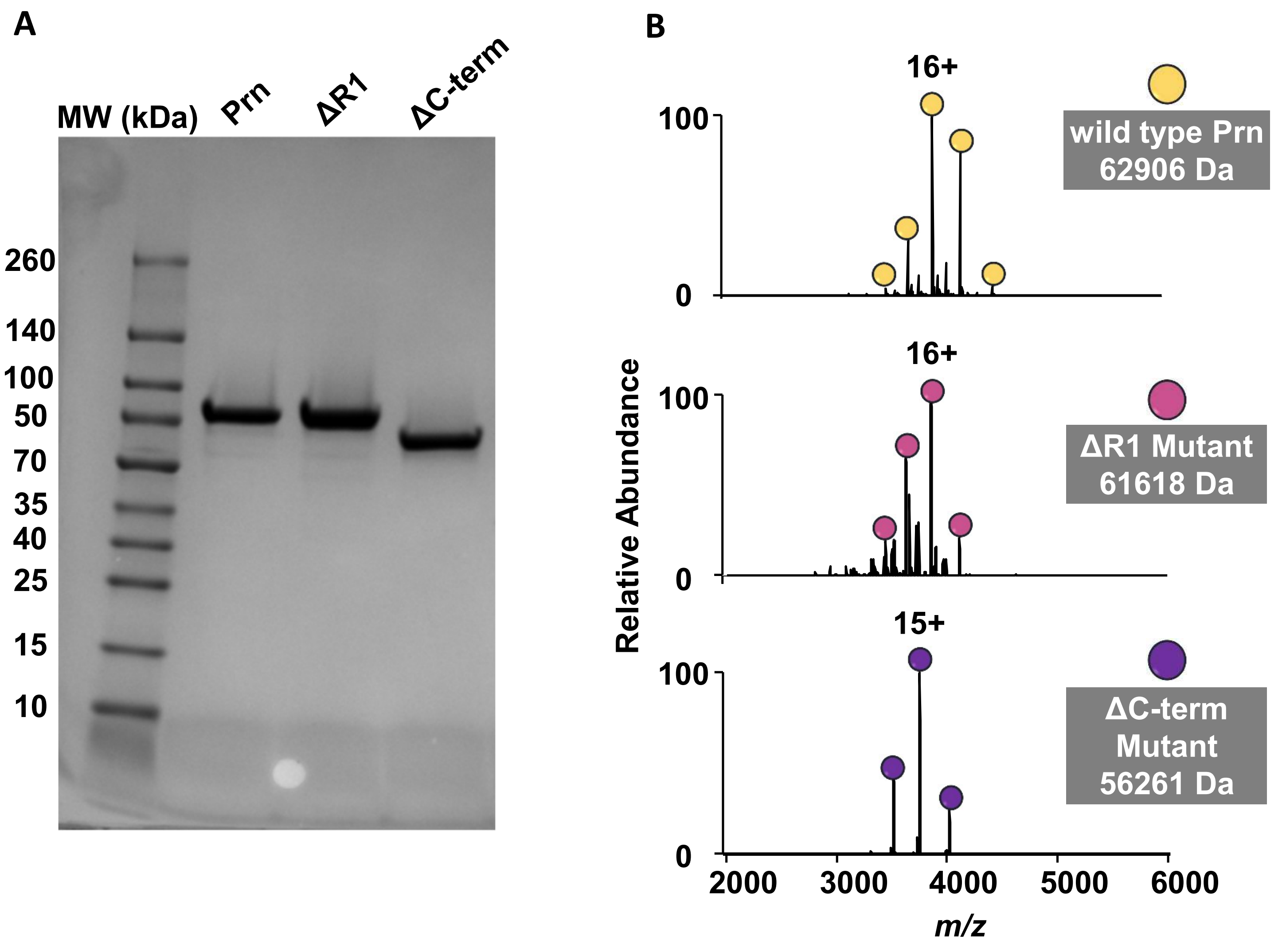

Figure S5. Screening of the interaction of therapeutic mAbs targeting Prn using native MS. MS1 spectra were obtained for solutions containing 2 µM of one antibody (A: NC, B: 2E9, C: 2B1, D: 1E7) and 2 µM of Prn in 250 mM ammonium acetate with 0.06% of C10E5. MS data was collected using positive polarity with a spray voltage of 0.9-1.1 kV, in-source trapping -100 to -150 V depending on the antibody, and 9 trapping gas, corresponding to a UHV gauge reading of around 9 × 10^{-10} to 9.5 × 10^{-10} mbar. The isotype NC antibody did not interact with Prn, as evidenced by the absence of peaks related to complex formation. In contrast, other antibodies resulted in formation of 1•1 and 1•2 mAb●Prn complexes in various charge states.

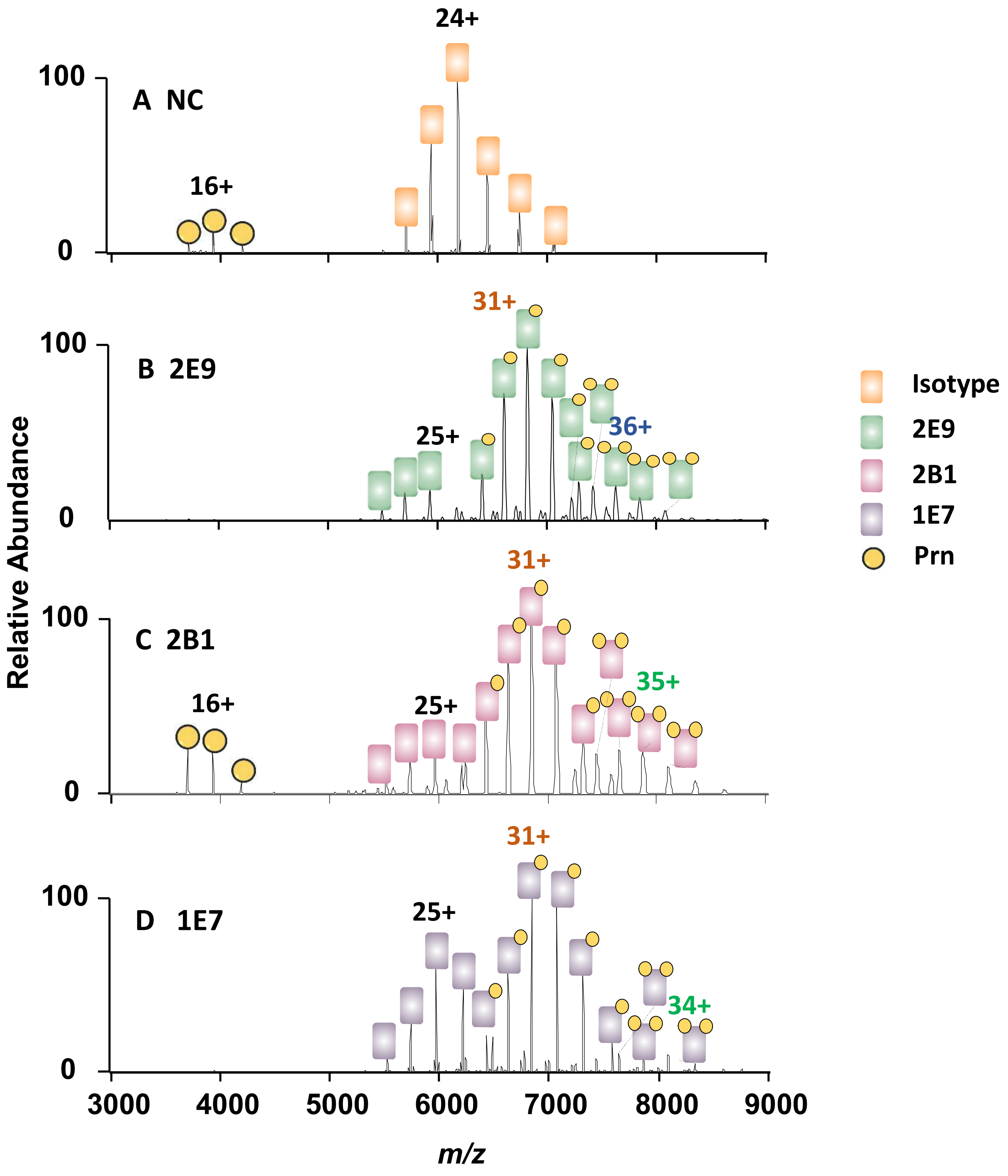

Figure S6: Application of the SLOMO method for studying the interaction of Prn and 1E7 mAb. Representative ESI mass spectra acquired at different times after mixing solution 1 (0.1 μM Prn + 1 μM 1E7 mAb) and solution 2 (1 μM Prn + 1 μM 1E7 mAb). The slow diffusion and lack of full equilibration, likely due to the large molecular sizes of the interaction partners, demonstrate the impracticality of this method for full length antibody–antigen systems.

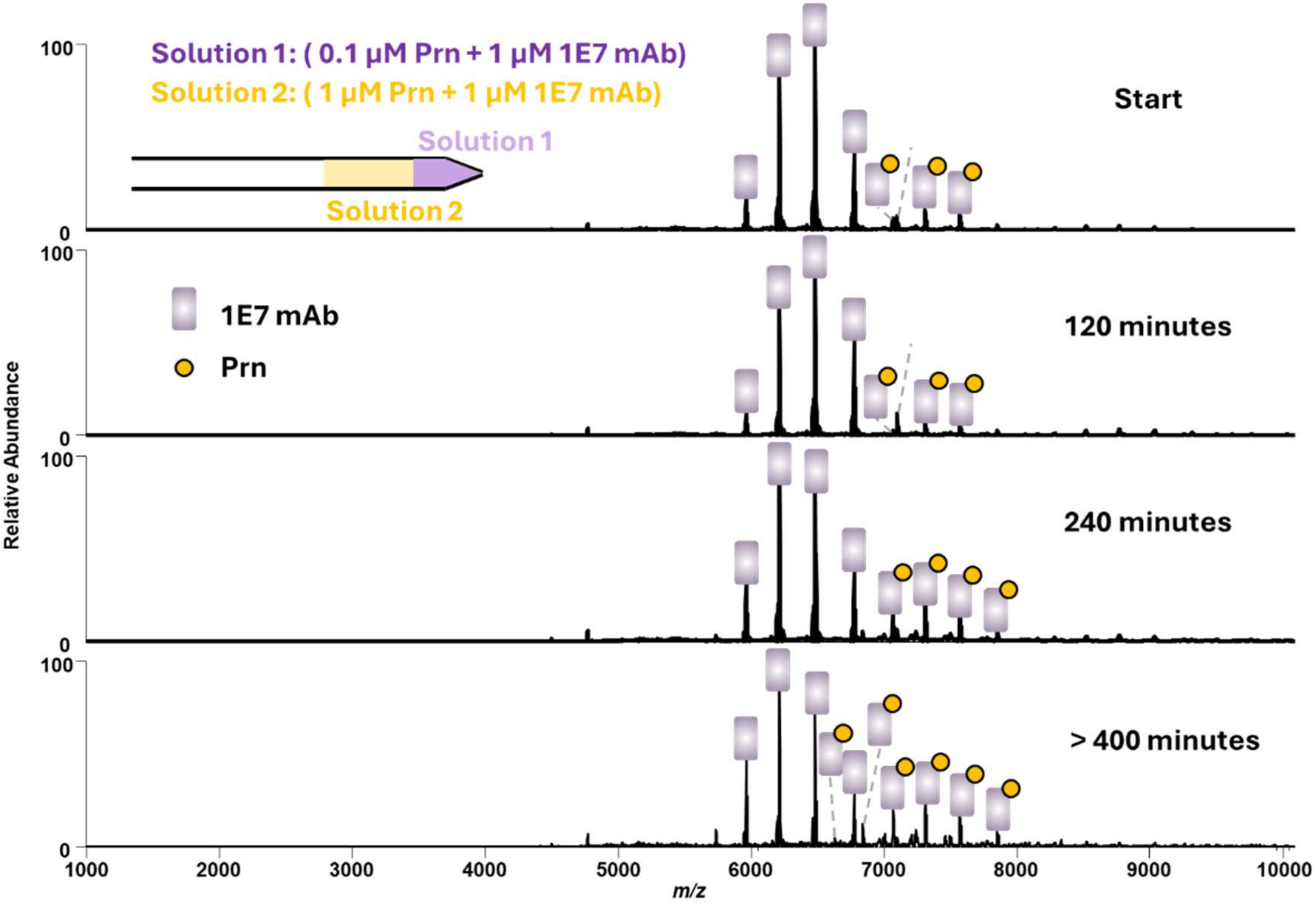

Figure S7. Equilibrium constant (K_d) measurements using biolayer interferometry for different mAbs targeting Prn. Data collected using anti-mice Fc BLI biosensors coated with 10 nM of mAbs. Then each biosensor was dipped into six serial dilutions of Prn, starting from an initial concentration of 50 nM, with 1:2 dilutions (corresponding to Prn concentrations of 50 nM, 25 nM, 12.5 nM, 6.25 nM, 3.125 nM, and 1.56 nM) to allow association followed by incubating the biosensors in kinetic buffer for five minutes to monitor the dissociation phase. The start of the dissociation phase is marked by a vertical red line in each graph. The binding profiles exhibit rapid association and slow dissociation, consistent with high-affinity interactions. These results agree with previously reported nanomolar dissociation constants for these mAbs.[2]

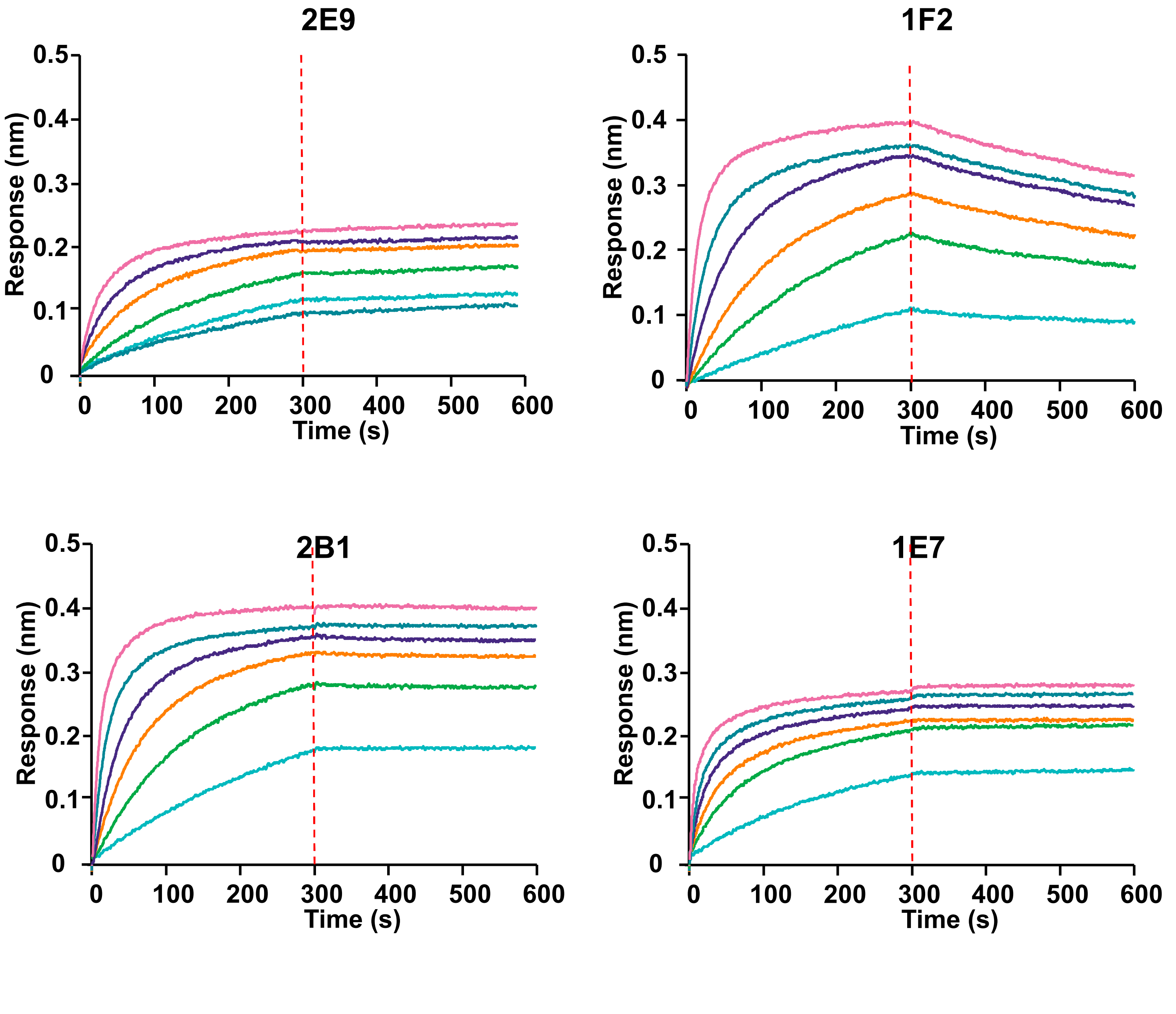

Figure S8. Titration experiment of 2E9 with Prn using native MS. (A) ESI mass spectra obtained after incubating various concentrations of Prn (0.5, 1, 2, or 4 μM Prn) with 2 μM 2E9 antibody in 250 mM ammonium acetate with 0.06% of C10E5. The corresponding deconvoluted spectra are shown on the right. The spectra display an increase in relative abundances of both 2E9•Prn and 2E9•$[Prn]_2$ complexes as the Prn concentration increases. (B) MS1 spectrum of a solution containing 8 uM Prn with 2 uM of 2E9 antibody, confirming that a further increase of Prn concentration does not lead to the formation of higher stoichiometry complexes.

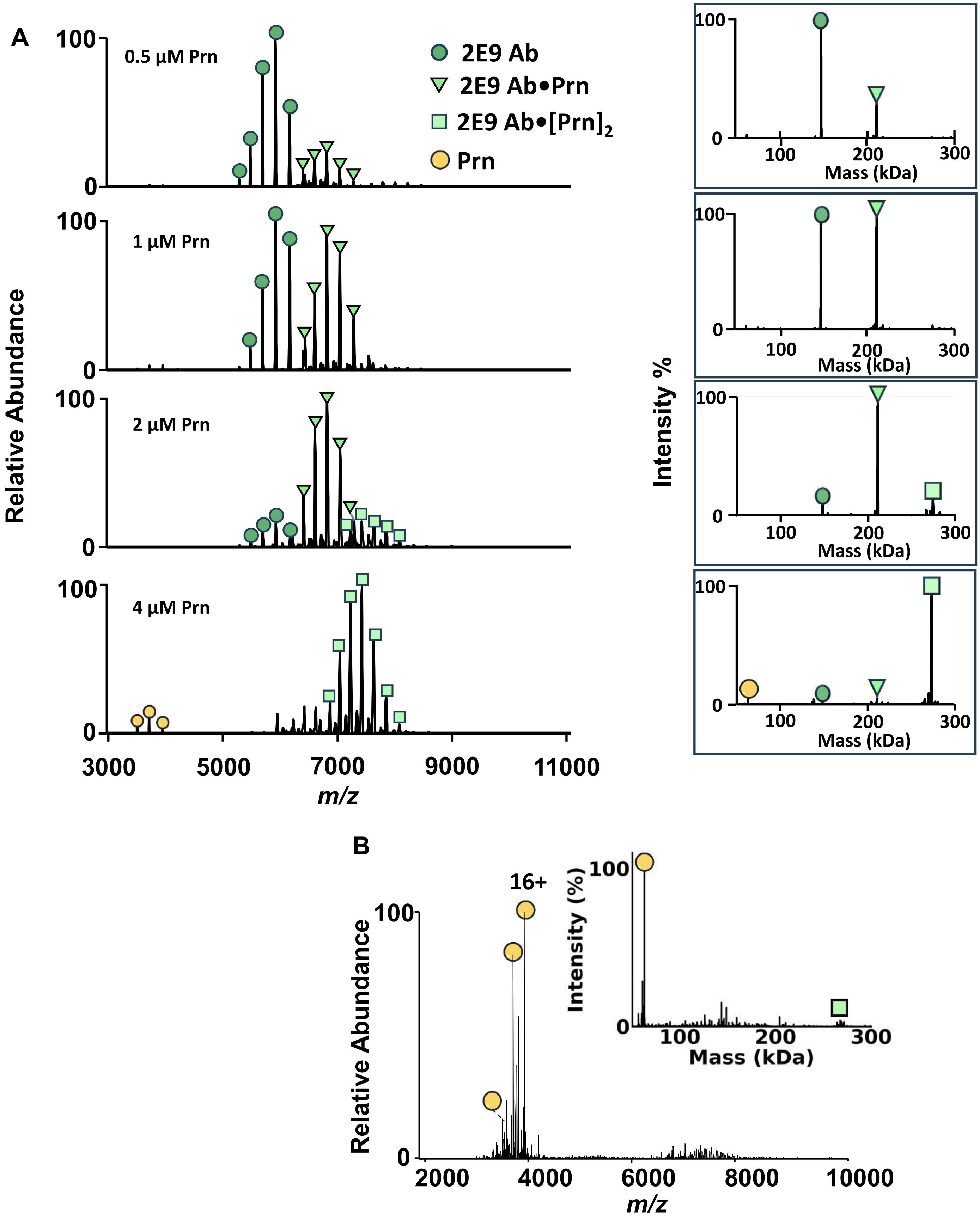

Figure S9. Titration experiment of 1F2 using native MS. (A) ESI mass spectra obtained after incubating various concentrations of Prn (0.5, 1, 2, or 4 µM Prn) with 2 µM 1F2 antibody in 250 mM ammonium acetate with 0.06% of C10E5. The corresponding deconvoluted spectra are shown on the right. The spectra display an increase in relative abundances of both 1F2•Prn and 1F2•$[Prn]_2$ complexes as the Prn concentration increases. (B) MS1 spectrum of a solution containing 8 uM Prn with 2 uM of 1F2 antibody confirming that a further increase of Prn concentration does not lead to the formation of higher stoichiometry complexes.

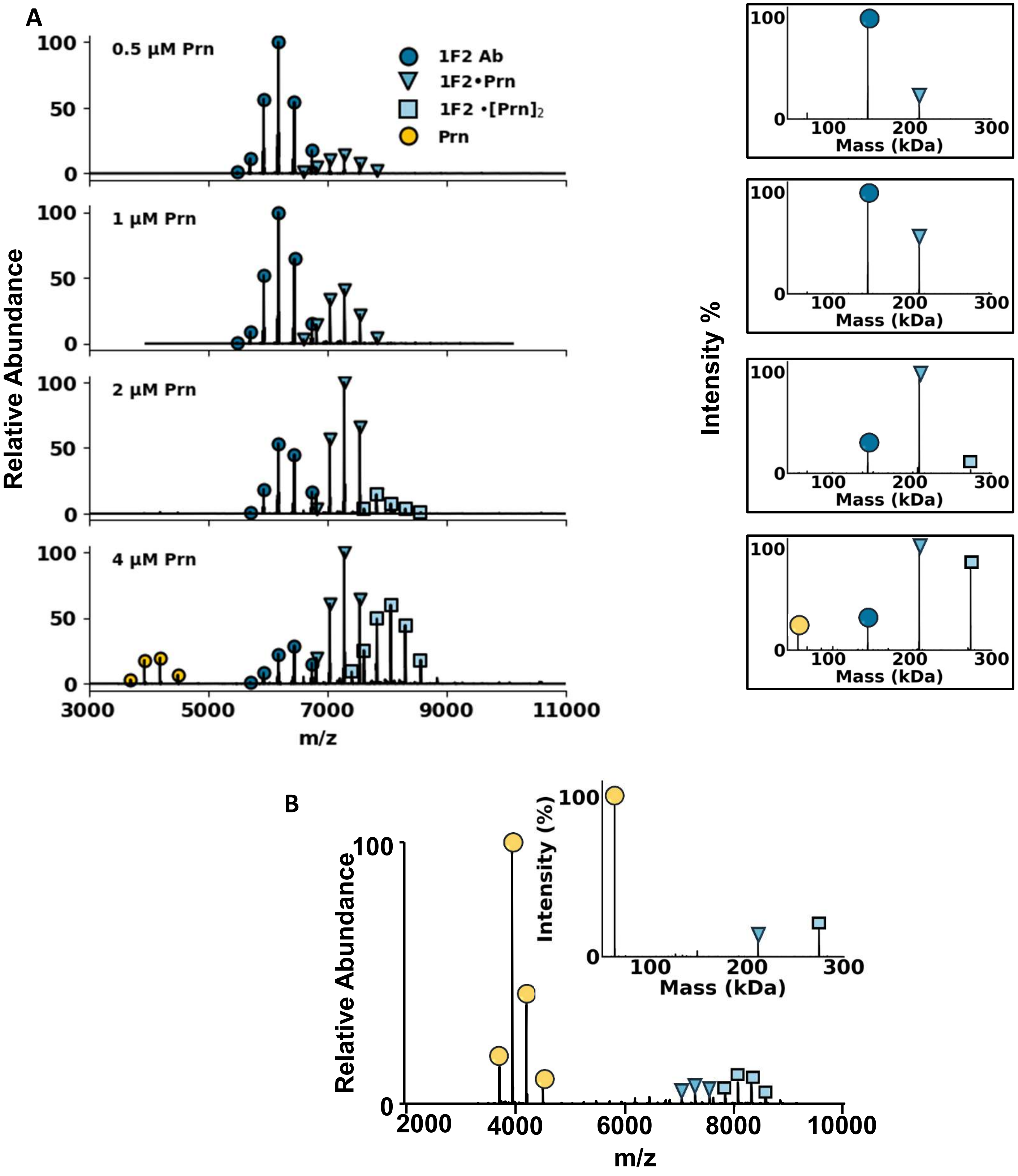

Figure S10. Titration experiment of 2B1 using native MS. (A) ESI mass spectra obtained after incubating various concentrations of Prn (0.5, 1, 2, or 4 µM Prn) with 2 µM 2B1 antibody in 250 mM ammonium acetate with 0.06% of C10E5. The corresponding deconvoluted spectra are shown on the right. The spectra display an increase in relative abundances for both 2B1•Prn and 2B1•$[Prn]_2$ complexes as the Prn concentration increases. (B) Bar graph showing the relative intensities of the 2B1•Prn and 2B1•$[Prn]_2$ complexes at different Prn concentrations. (C) MS1 spectrum of a solution containing 8 uM Prn with 2 uM of 2B1 antibody confirming that a further increase of Prn concentration does not lead to the formation of higher stoichiometry complexes.

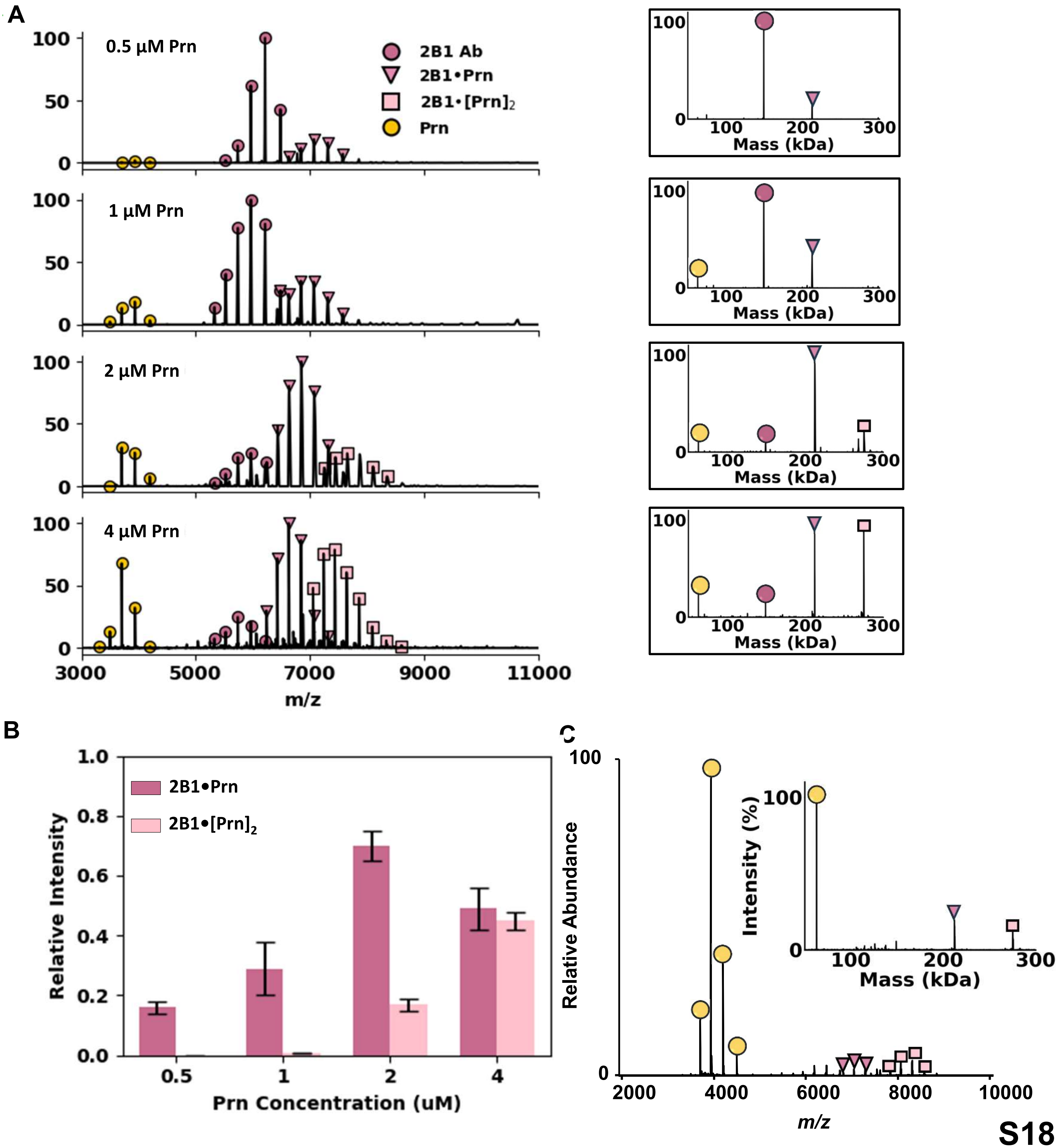

Figure S11. Titration experiment of 1E7 using native MS. (A) ESI mass spectra obtained after incubating various concentrations of Prn (0.5, 1, 2, or 4 µM Prn) with 2 µM 1E7 antibody in 250 mM ammonium acetate with 0.06% of C10E5. The corresponding deconvoluted spectra are shown on the right. The spectra display an increase in relative abundances for both 1E7•Prn and 1E7•[Prn]$_2$ complexes as the Prn concentration increases. (B) Bar graph showing the relative intensities of the 1E7•Prn and 1E7•[Prn]$_2$ complexes at different Prn concentrations. (C) MS1 spectrum of a solution containing 8 uM Prn with 2 uM of 1E7 antibody confirming that a further increase of Prn concentration does not lead to the formation of higher stoichiometry complexes.

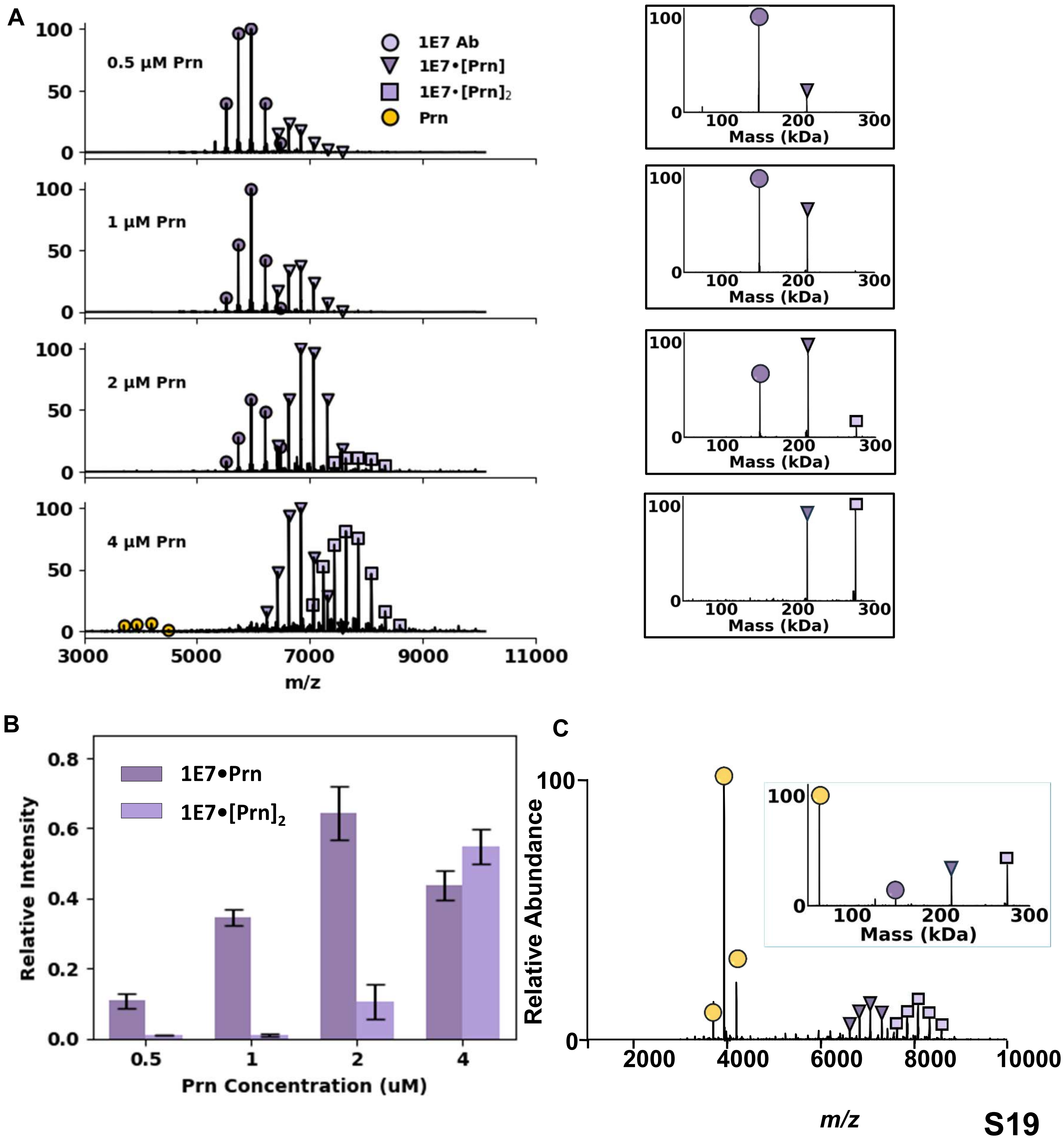

Figure S12. Comparison of antibody–antigen complex stoichiometries determined by native MS. Gaussian fits to deconvoluted mass spectra obtained from ESI-MS of 2 µM antibody solutions incubated with 4 µM Prn in 250 mM ammonium acetate containing 0.06 % C10E5 detergent. (A) 1F2 forms both 1:1 and 1:2 mAb•Prn complexes, whereas (B) 2E9 predominantly yields the 1:2 mAb•$[Prn]_2$ complex. The results illustrate distinct stoichiometric distributions among antibodies, with 2E9 exhibiting a higher fraction of the bivalent complex under these conditions.

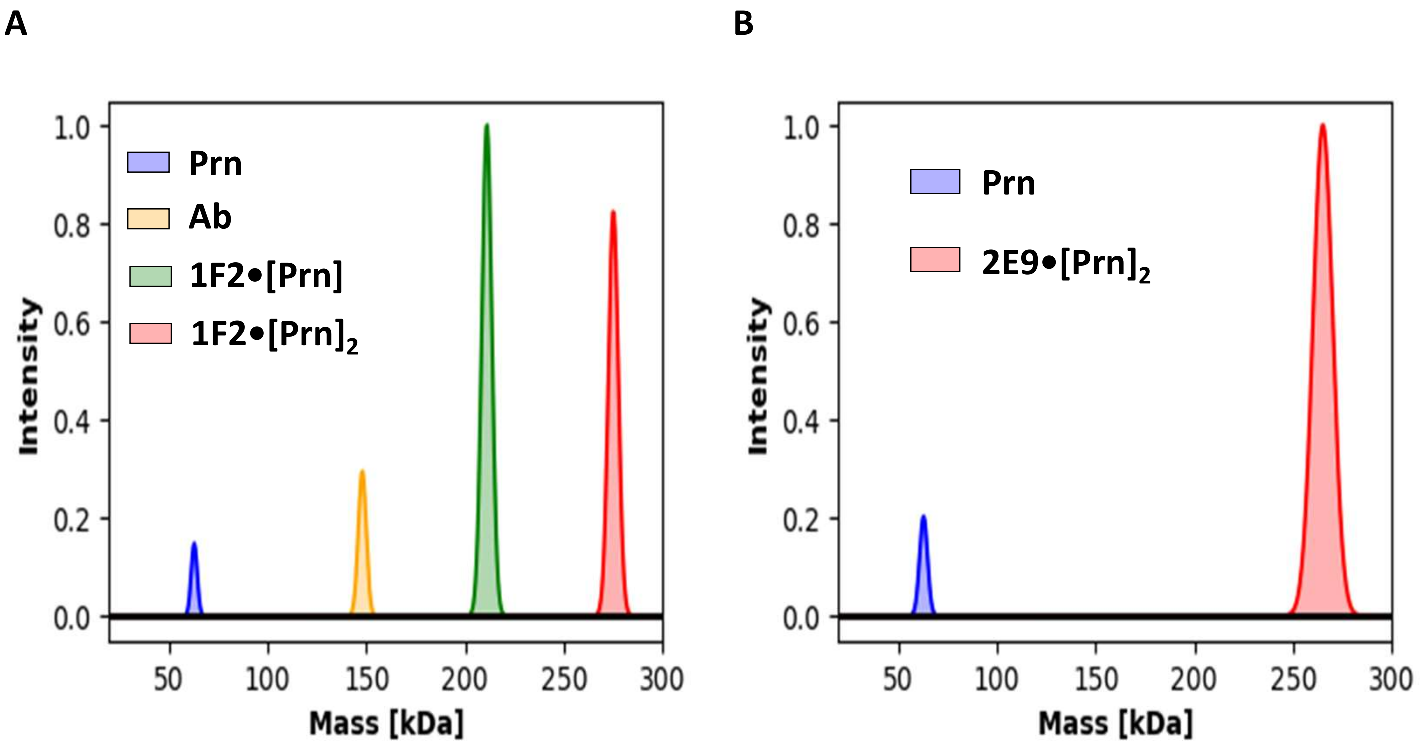

Figure S13. Screening of simultaneous binding of different mAb pairs targeting different Prn epitopes. (A-C) MS1 spectra obtained from incubation of 5 µM pairs of antibodies (1E7/2E9, 2B1/2E9 and 1E7/1F2), respectively, with equimolar concentration of Prn. Figures on the right show Gaussian fits to the deconvoluted mass spectra. (D-F) Schematic representations of Prn+2E9-Fab+1E7-Fab, Prn+2B1-Fab+2E9-Fab and Prn+1E7-Fab+1F2-Fab, respectively, with crystal structure Prn shaded gray (1DAB), and AlphaFold-predicted 2E9-Fab shaded green, 2B1-Fab shaded pink, 1F2-Fab shaded blue and 1E7-Fab shaded purple, all shown as molecular surfaces. The presence of ion peaks corresponding to complexes containing Prn and two Abs indicates that Abs that bind to distant epitopes can simultaneously bind to the Prn antigen.

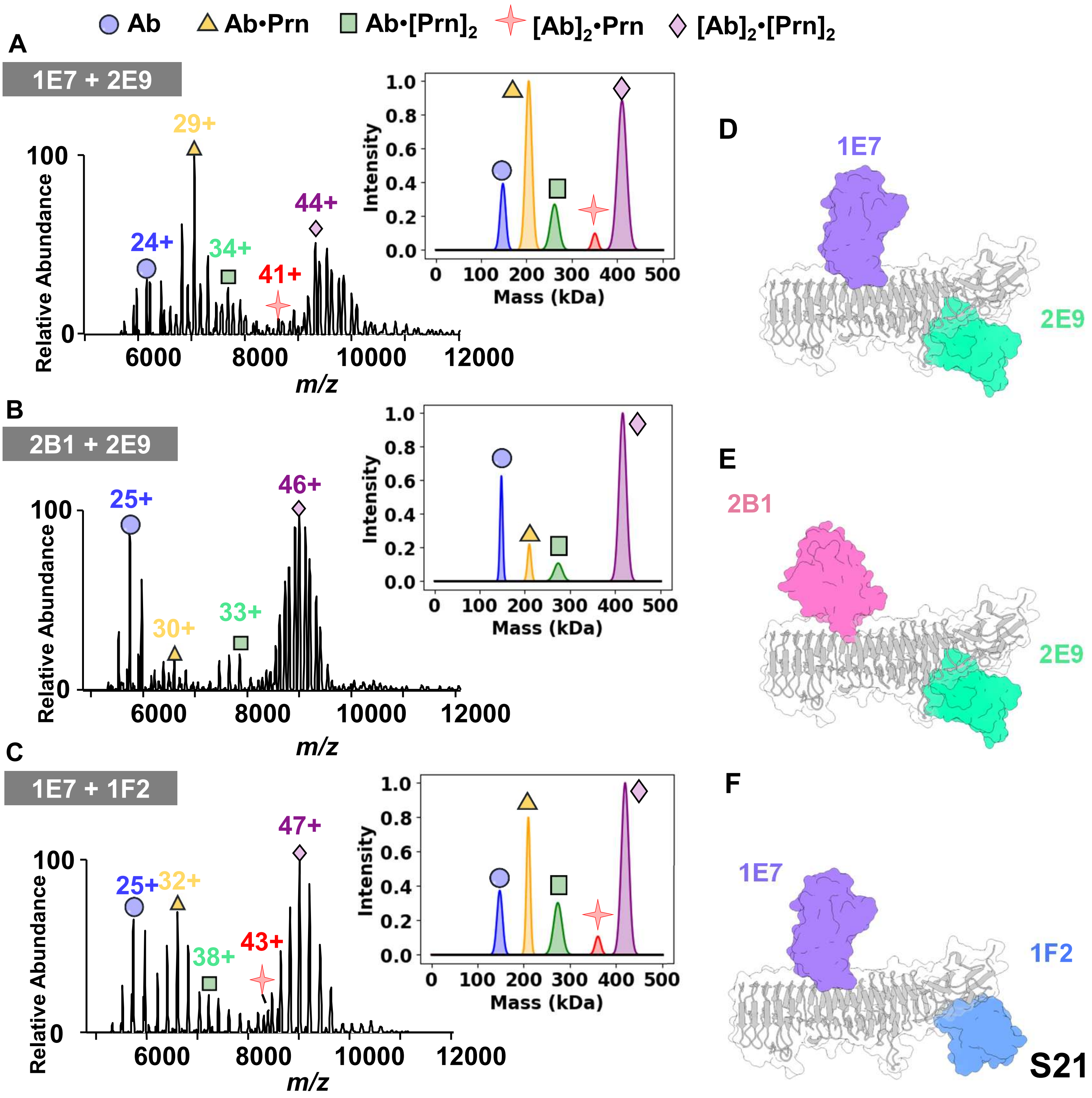

Figure S14. Screening of simultaneous binding of different mAb pairs targeting different Prn epitopes. (A) MS1 spectrum obtained from incubation of 5 μM 1F2/2E9 with equimolar concentration of Prn. B) Figure shows Gaussian fits to deconvoluted mass spectrum. (C) Schematic representation of Prn+1F2-Fab+2E9-Fab with crystal structure Prn shaded gray (1DAB) and AlphaFold-predicted 2E9-Fab shaded green and 1F2-Fab shaded blue, all shown as molecular surfaces. Simultaneous binding of 2E9 and 1F2 is suppressed, resulting in low intensity peaks for the $[Ab]_2$●Prn complexes.

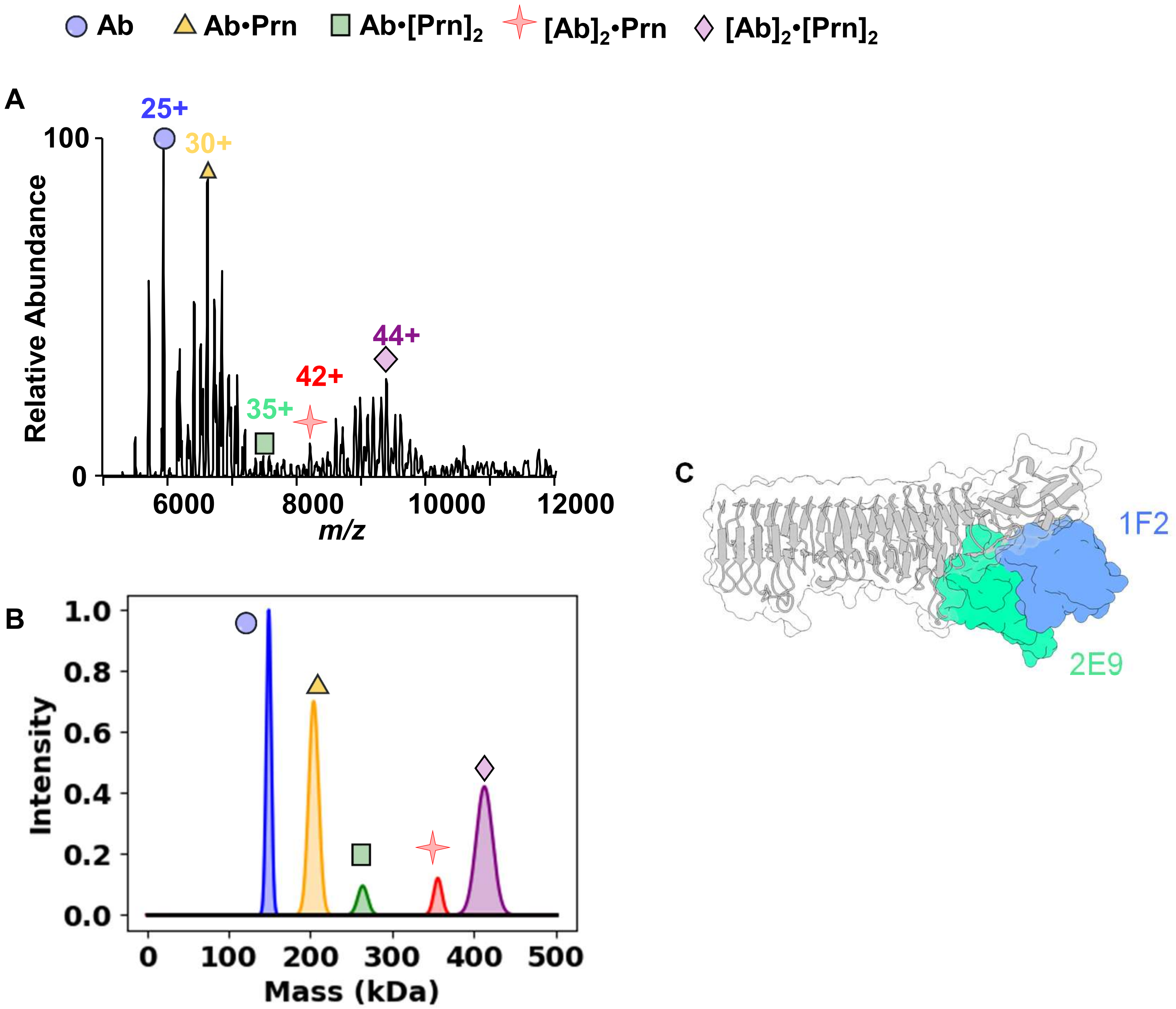

Figure S15. Evaluation of the thermal stability of different antibodies using vT-ESI MS. (A,B,C,D) MS1 spectra illustrating the shift in average charge state distribution with increasing solution temperature, ranging from 20°C (top panels) to 60 °C (bottom panels). The spectra were obtained from solutions contain 2 μM of antibody: A: 2E9 green, B; 2B1 pink, C: 1F2 blue and D: 1E7 purple. (E) Plot of the average charge state of each antibody as a function of solution temperature.

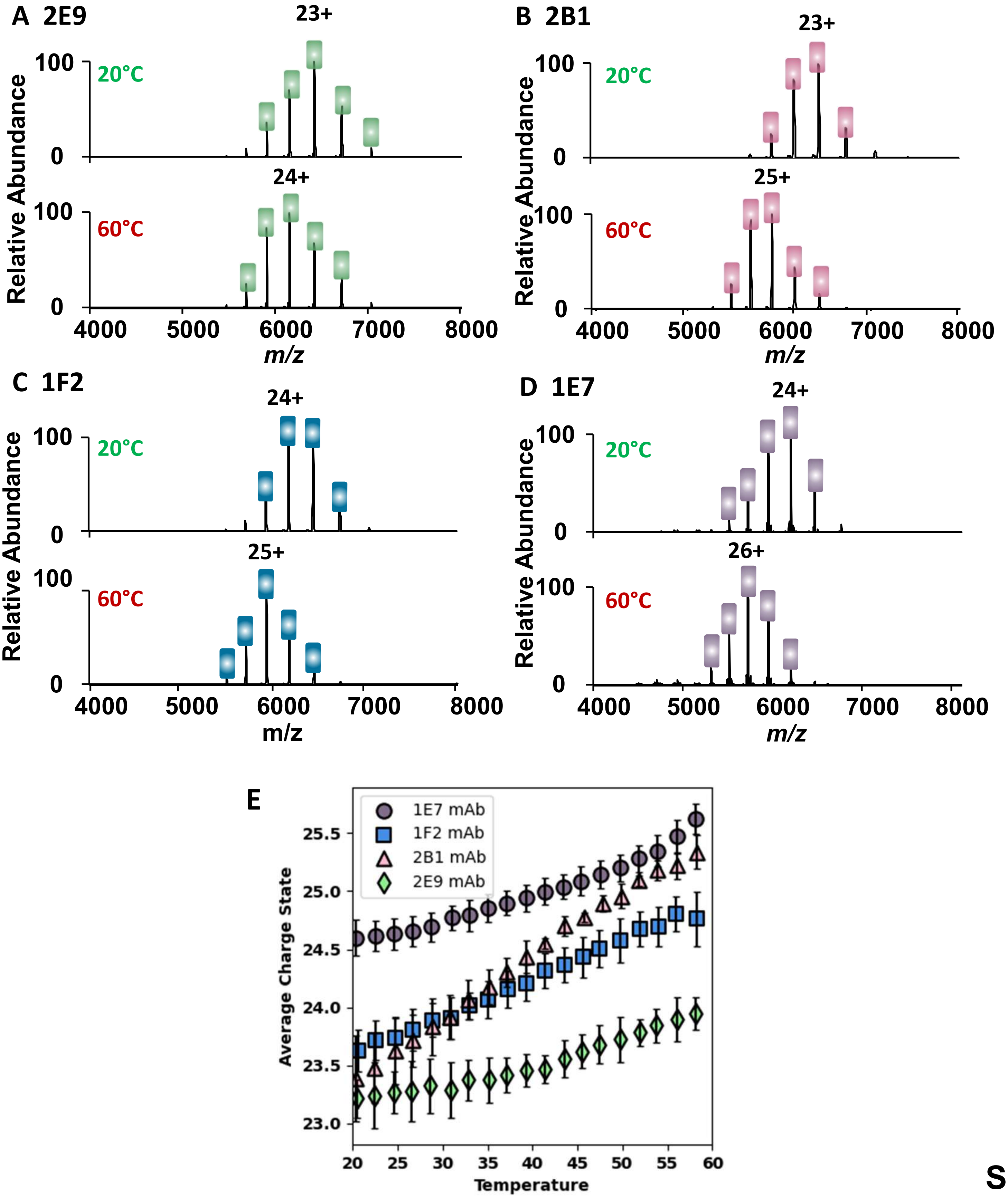

Figure S16. Evaluation of the thermal induced unfolding of different antibody•Prn complexes using vT-ESI native MS. (A,B,C) MS1 spectra illustrating the shift in average charge state distribution with increasing solution temperature, ranging from 20°C (top panels) to 60 °C (bottom panels). The spectra were obtained for solutions containing 2 µM of an antibody (A; 2E9 green, B: 2B1 pink, C: 1E7 purple) with 2 µM of Prn in 250 mM ammonium acetate with 0.06% C10E5.

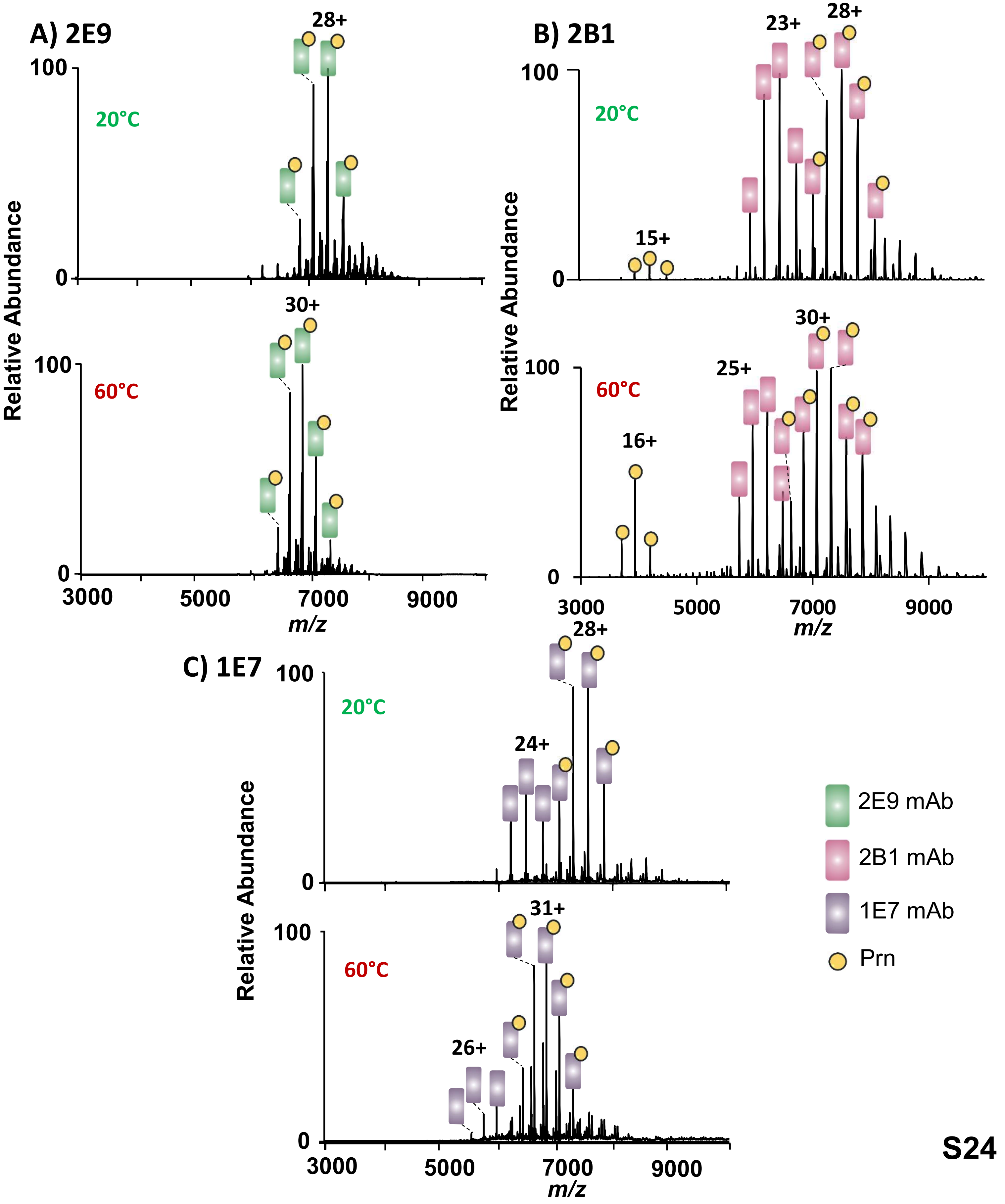

Figure S17. Variable temperature-ESI-MS for evaluation of thermal stability of different mAb•Prn complexes at elevated solution temperature of 70°C. MS1 spectra demonstrate the thermal induced dissociation of different mAb•Prn complexes at high solution temperature. Notably, 2E9 shows remarkable stability; its complex remains intact and does not release the Prn antigen even at 70°C.

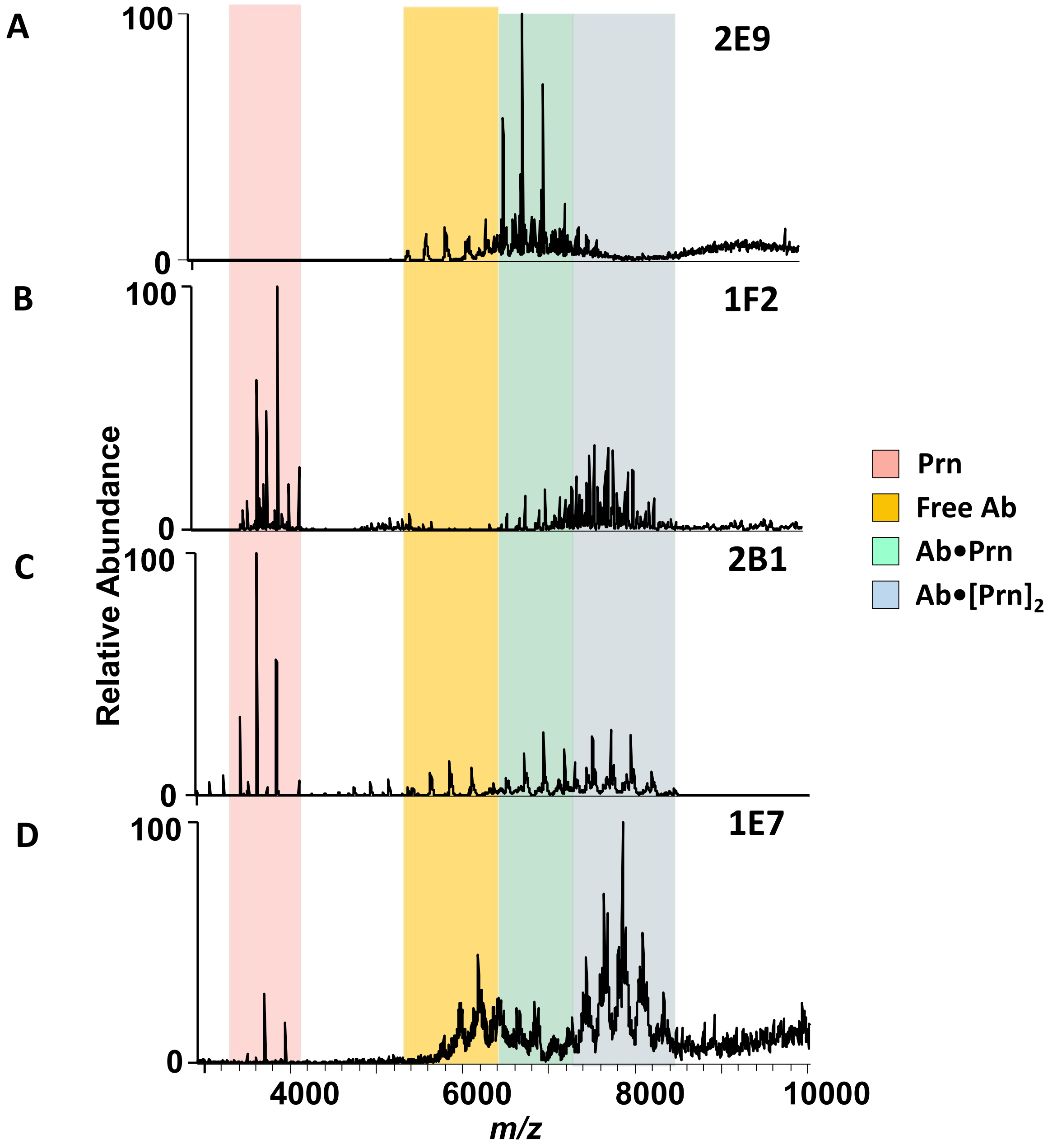

Figure S18. Sequence alignment of Prn, ΔR1 and ΔC-term mutants from *Bordetella Pertussis* generated with ClustalOmega

```
Prn        1    MGSSWSHPQFEKSSGASDWNNQSIVKTGERQHGIHIQGSDPGGVRTASGTTIKVSGRQAQGILLENPAAELQFRNGSVTSSGQLSDDGIR  90
ΔR1        1    MGSSWSHPQFEKSSGASDWNNQSIVKTGERQHGIHIQGSDPGGVRTASGTTIKVSGRQAQGILLENPAAELQFRNGSVTSSGQLSDDGIR  90
ΔC-term    1    MGSSWSHPQFEKSSGASDWNNQSIVKTGERQHGIHIQGSDPGGVRTASGTTIKVSGRQAQGILLENPAAELQFRNGSVTSSGQLSDDGIR  90

Prn        91   RFLGTVTVKAGKLVADHATLANVGDTWDDDGIALYVAGEQAQASIADSTLQGAGGVQIERGANVTVQRSAIVDGGLHIGALQSLQPEDLP  180
ΔR1        91   RFLGTVTVKAGKLVADHATLANVGDTWDDDGIALYVAGEQAQASIADSTLQGAGGVQIERGANVTVQRSAIVDGGLHIGALQSLQPEDLP  180
ΔC-term    91   RFLGTVTVKAGKLVADHATLANVGDTWDDDGIALYVAGEQAQASIADSTLQGAGGVQIERGANVTVQRSAIVDGGLHIGALQSLQPEDLP  180

Prn        181  PSRVVLRDTNVTAVPASGAPAAVSVLGASELTLDGGHITGGRAAGVAAMQGAVVHLQRATIRRGDAPAGGGVPGGAVPGGAVPGGFGPGG  270
ΔR1        181  PSRVVLRDTNVTAVPASGAPAAVSVLGASELTLDGGHITGGRAAGVAAMQGAVVHLQRATIRRGDAPAGGG---------------GSGG  255
ΔC-term    181  PSRVVLRDTNVTAVPASGAPAAVSVLGASELTLDGGHITGGRAAGVAAMQGAVVHLQRATIRRGDAPAGGAVPGGAVPGGAVPGGFGPGG  270

Prn        271  FGPVLDGWYGVDVSGSSVELAQSIVEAPELGAAIRVGRGARVTVSGGSLFAPHGNVIETGGARRFAPQAAPLSITLQAGAHAQGKALLYR  360
ΔR1        256  GGSVLDGWYGVDVSGSSVELAQSIVEAPELGAAIRVGRGARVTVSGGSLSAPHGNVIETGGARRFAPQAAPLSITLQAGAHAQGKALLYR  345
ΔC-term    271  FGPVLDGWYGVDVSGSSVELAQSIVEAPELGAAIRVGRGARVTVSGGSLSAPHGNVIETGGARRFAPQAAPLSITLQAGAHAQGKALLYR  360

Prn        361  VLPEPVKLTLTGGADAQGDIVATELPSIPGTSIGPLDVALASQARWTGATRAVDSLSIDNATWVMTDNSNVGALRLASDGSVDFQQPAEA  450
ΔR1        346  VLPEPVKLTLTGGADAQGDIVATELPSIPGTSIGPLDVALASQARWTGATRAVDSLSIDNATWVMTDNSNVGALRLASDGSVDFQQPAEA  435
ΔC-term    361  VLPEPVKLTLTGGADAQGDIVATELPSIPGTSIGPLDVALASQARWTGATRAVDSLSIDNATWVMTDNSNVGALRLASDGSVDFQQPAEA  450

Prn        451  GRFKVLTVNTLAGSGLFRMNVFADLGLSDKLVVMQDASGQHRLWVRNSGSEPASANTLLLVQTPRGSAATFTLANKDGKVDIGTYRYRLA  540
ΔR1        436  GRFKVLTVNTLAGSGLFRMNVFADLGLSDKLVVMQDASGQHRLWVRNSGSEPASANTLLLVQTPLGSAATFTLANKDGKVDIGTYRYRLA  525
ΔC-term    451  GRFKVLTVNTLAGSGLFRMNVFADLGLSDKLVVMQDASGQHRLWVRNSGSEPASANTLLLVQTPLGSAATFTLANKDGKVDIGTYRYRLA  540

Prn        541  ANGNGQWSLVGAKAPPAPKPAPQPG---PQPPQPQPEAPAPQPPAGRELSAAANAAVNTGGVGLASTLWYAESNALSKRLGEL  620
ΔR1        526  ANGNGQWSLVGAKAPPAPKPAPQPGPQPPQPPQPQPEAPAPQPPAGRELSAAANAAVNTGGVGLASTLWYAESNALSKRLGEL  608
ΔC-term    541  ANGNGQWSLVGAKAPP-------------------------------------------------------------------  556
```

Figure S19. (A) MS1 spectrum obtained for a solution containing 2 μM of both ΔR1 and PeM-4 Ab in 250 mM ammonium acetate with 0.06% C10E5 detergent (B) MS1 spectrum obtained for a solution containing 2 μM of both Prn and PeM-4 Ab in 250 mM ammonium acetate with 0.06% C10E5 detergent. The insets highlight the Gaussian fits for the deconvoluted mass spectra.

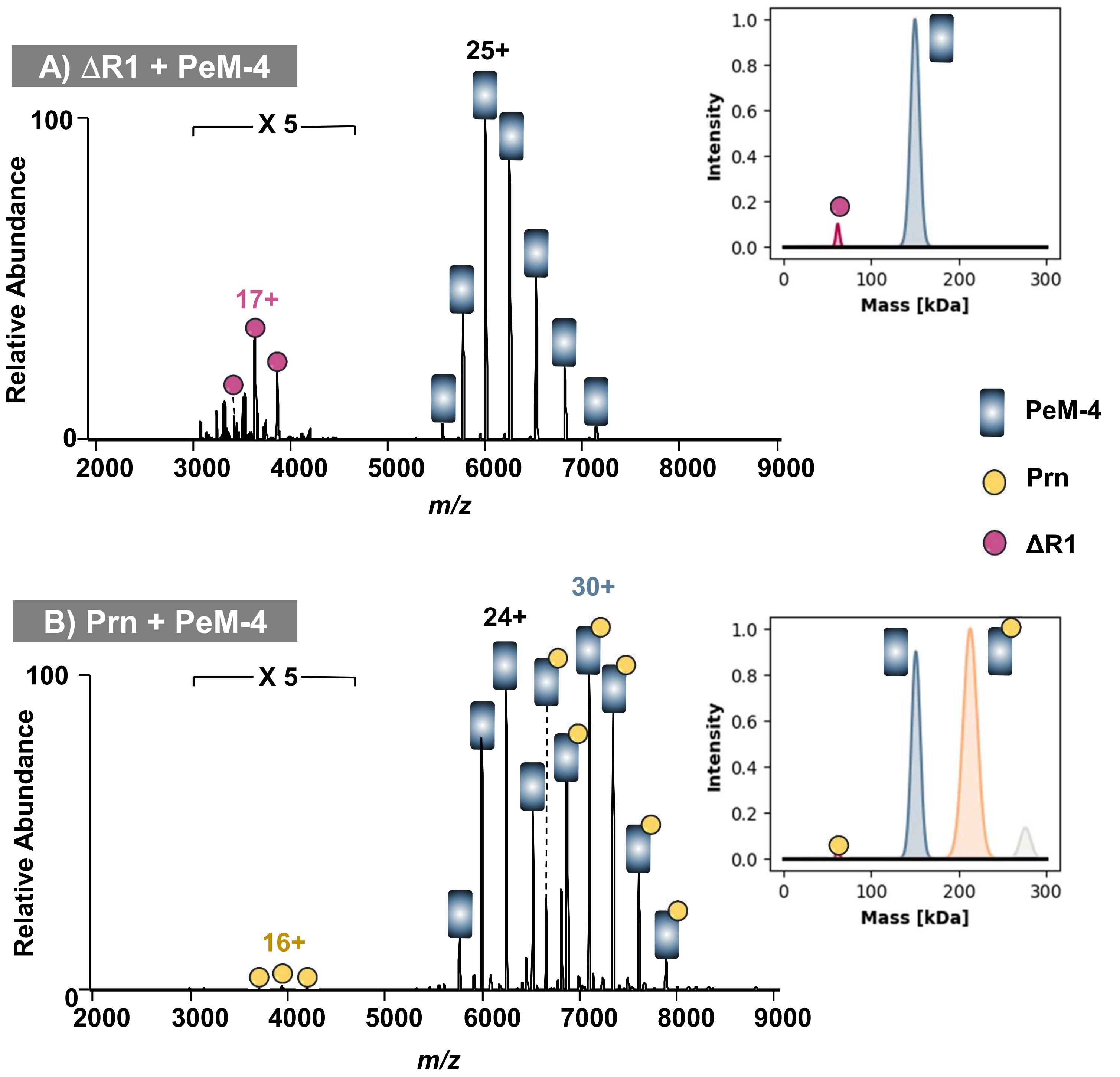

Figure S20. Monitoring interactions of high concentrations of ΔR1 and ΔC-term mutants with specific monoclonal antibodies. (A) Mass spectra of solutions containing 4 μM ΔR1 mutant incubated with 2 μM R1-specific monoclonal antibody PeM-4, and (B) 4 μM ΔC-term mutant incubated with 2 μM C-term-specific monoclonal antibody PeM-19. The experiments demonstrate the absence of binding interactions even at twice the protein concentration.

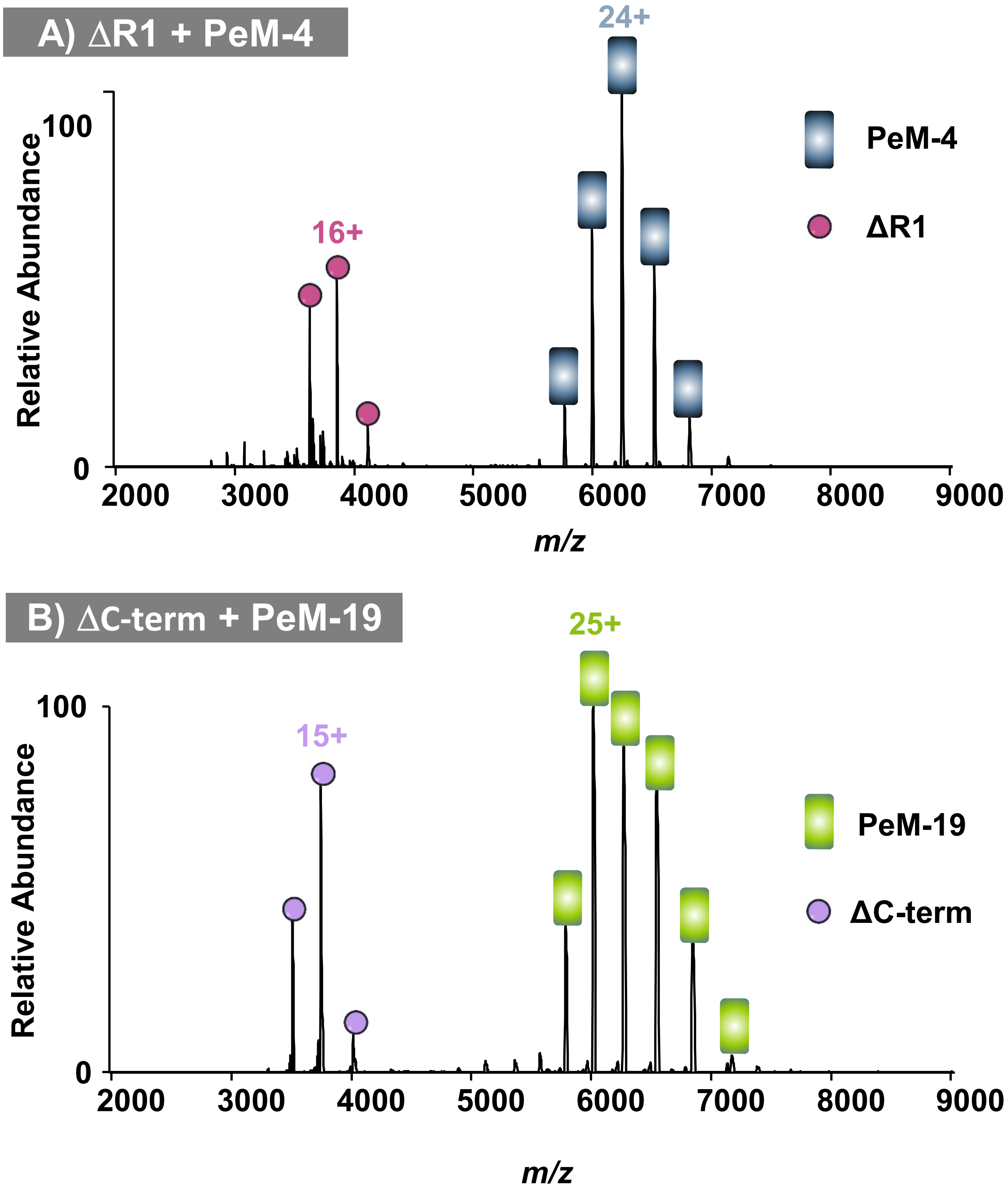

Figure S21. ESI MS1 spectra of monoclonal antibodies targeting different Prn epitopes. Each antibody was buffer exchanged into 200 mM ammonium acetate and analyzed using the UHMR mass spectrometer with 1.0 kV to 1.1 kV as spray voltage, 6 trapping gas and -100V to -120V as desolvation voltage.

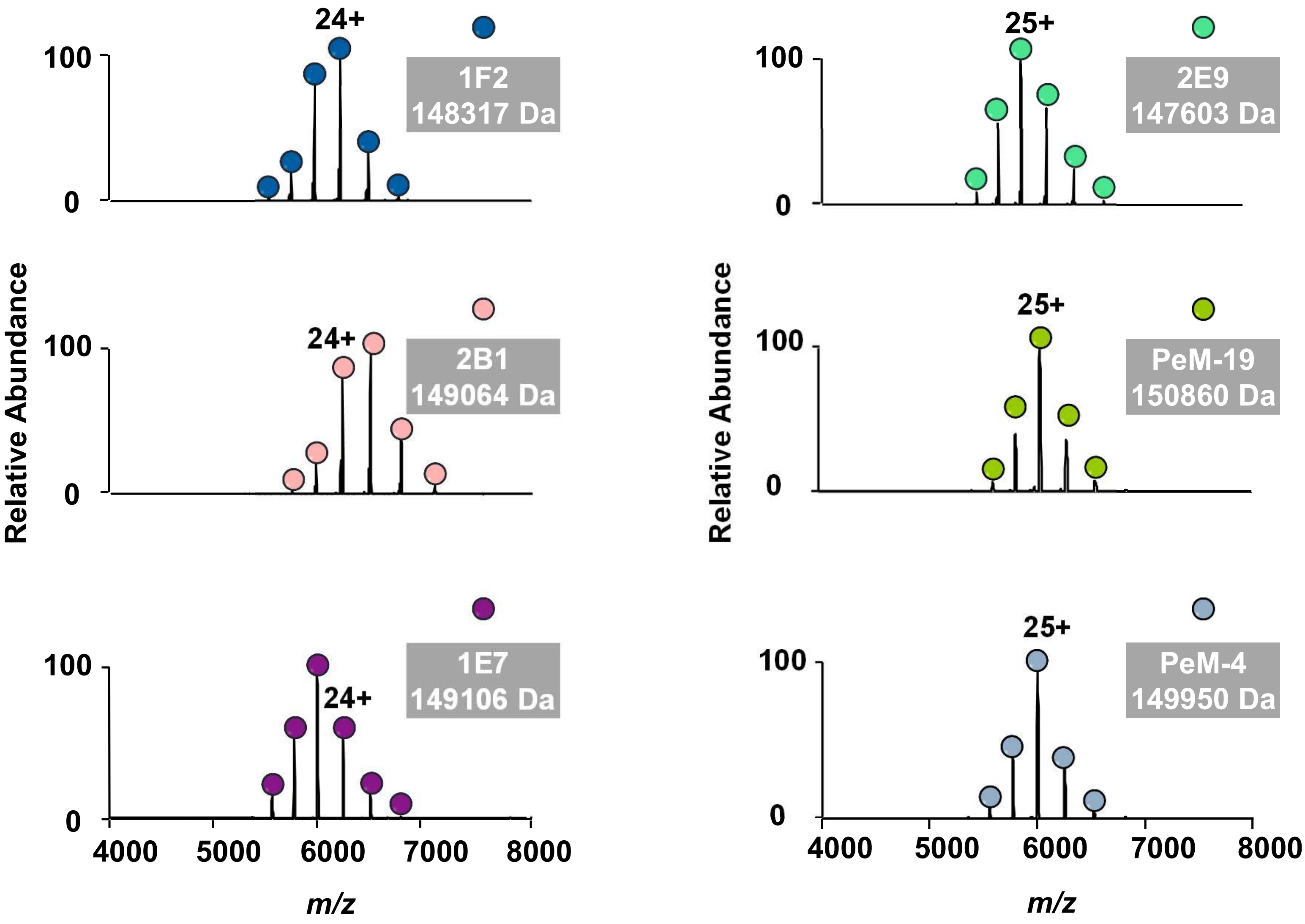

Figure S22. Analysis of deconvoluted mass spectra obtained from UniDec[2] using Gaussian fitting and simplification. Left panel represent deconvoluted MS1 spectrum obtained from UniDec. The middle panel refines this data further using Gaussian fitting, highlighting significant peaks that correspond to different protein or protein complexes. The right panel represents a simplified version of the spectrum, where only the most prominent peaks are retained for focused analysis of the key molecular components.

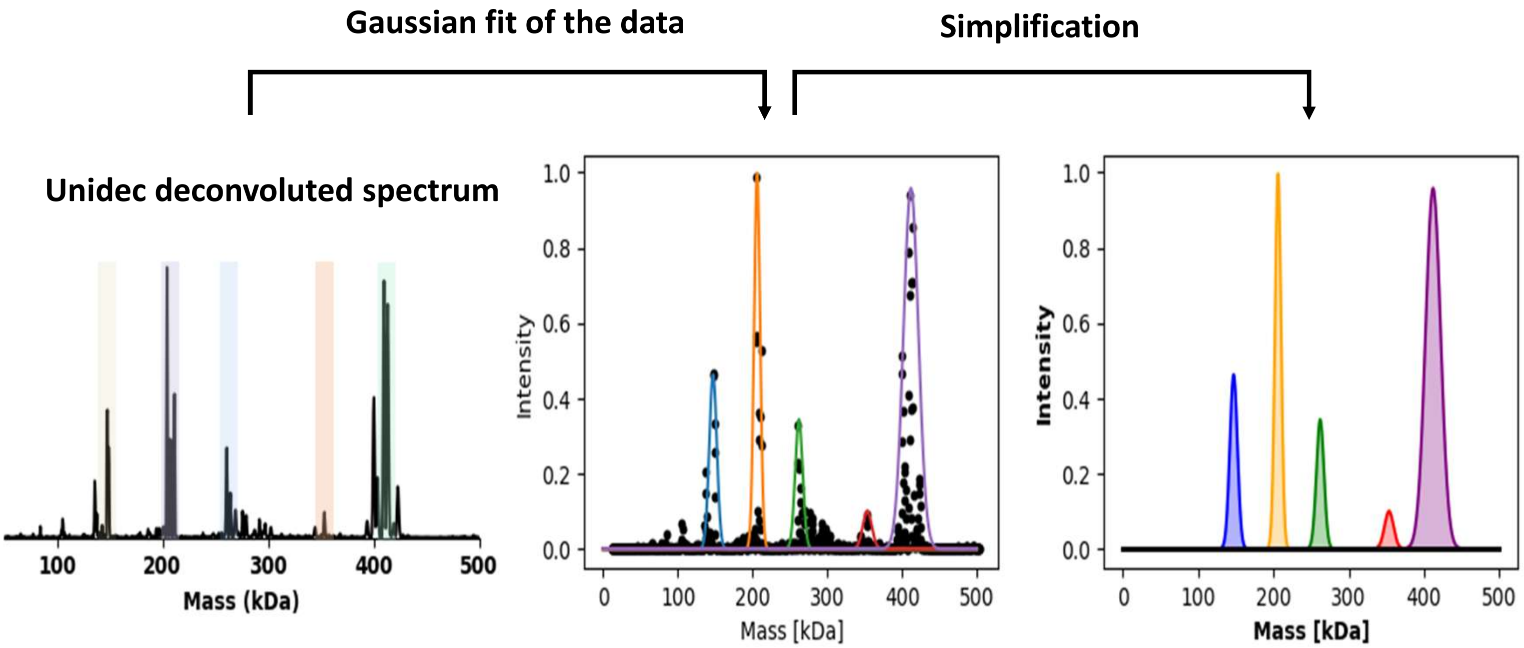

- **Antibody and Fab expression, purification, and quality control**

Antibodies binding to pertactin were purified as previously described[1]. In brief, ExpiCHO-S cells were transfected with mouse IgG2a antibody heavy chain and light chain plasmids[2] following manufacturer's instructions for the high-titer protocol. Heavy chain Fab plasmids contain a stop codon after the human CH1 domain . Plasmid heavy:light chain ratios of 1:3 and 1:1 were used for full-length antibody and Fab transfections, respectively. Mouse IgG2a was purified using a HiTrap® Protein A (Cytiva) on an AKTA Pure FPLC system (Cytiva). Human Fab was purified using CaptureSelect™ CH1-XL Affinity Matrix (Thermofisher) using the same FPLC system. Purified protein was buffer exchanged into PBS (Amicon; Millipore Sigma) and stored at -80°C. Protein quality was assessed with analytical size exclusion Superdex 200 (AKTA; Cytiva) and SDS-PAGE (4-20% gradient gel; Bio-Rad). The identity of the antibodies was confirmed using native mass spectrometry after buffer exchange into 200 mM ammonium acetate (**Figure S22**).

- **Pertactin expression, purification, and quality control**

We expressed pertactin and its variants as inclusion bodies based on previously described methods.[3] The inclusion body pellet was washed three times with 20 mL of 50 mM Tris, 5 mM EDTA, 2M Urea, 2% Triton X-100, pH 8.0, followed by centrifugation for 20 min at 20,000 rpm. An additional three washes containing 50 mM Tris, pH 8.0 were employed to remove the 2% Triton X-100. The inclusion body was stored at -20°C until use. To purify pertactin, 300 mg of the inclusion body was resuspended in urea buffer (8 M urea, 50 mM Tris, 100 mM NaCl, 0.2 mM $CaCl_2$, pH 8.0) to a concentration of 10 mg/mL, then centrifuged at 20,000 rpm for 20 min. The supernatant was diluted to 5 mg/mL in urea buffer and dialyzed in 1 L of buffer (50 mM Tris, 100 mM NaCl, 1 mM EDTA, pH 8.0) that was refreshed daily over 72 h. The dialyzed, refolded protein was centrifuged for 20,000 rpm for 20 min, filter sterilized, and purified using a Strep-Tactin®XT 4Flow (IBA) gravity column according to the manufacturer's protocol. The eluate was concentrated to no more than 2 mg/mL (Amicon; Millipore Sigma), filtered, and SEC purified in 1 mL aliquots (Superdex 200 on an AKTA Pure FPLC system; Cytiva). Purified pertactin was concentrated to no more than 2 mg/mL (Amicon; Millipore Sigma), filter sterilized, aliquoted, and flash-frozen for storage at -80°C. All produced pertactin was assessed for quality using indirect ELISA as previously described1 and SDS-PAGE (**Figure S4A**) (4-20% gradient gel; Bio-Rad). The identity of Prn and its mutants was finally confirmed using native mass spectrometry after buffer exchange into 300 mM ammonium acetate with 0.06% C10E5 detergent (**Figure S4B**).

References

1. Gupta R, Brunak S. Prediction of glycosylation across the human proteome and the correlation to protein function. Pac Symp Biocomput. 2002;:310-22. PMID: 11928486
2. Silva, R. P.; DiVenere, A. M.; Amengor, D.; Maynard, J. A. Antibodies Binding Diverse Pertactin Epitopes Protect Mice from Bordetella Pertussis Infection. *Journal of Biological Chemistry* 2022, *298* (3), 101715. https://doi.org/10.1016/J.JBC.2022.101715.
3. Kenneth Smith, Lori Garman, Jens Wrammert, Nai-Ying Zheng, J Donald Capra, Rafi Ahmed & Patrick C Wilson. Rapid generation of fully human monoclonal antibodies specific to a vaccinating antigen. Nature Protocol 4, 372–384 (2009).
